# Photoelectrosynthesis of adipic acid coupled with energy storage in an open-loop flow battery

**DOI:** 10.1093/nsr/nwag236

**Published:** 2026-04-22

**Authors:** Shanshan Zhang, Lan Luo, Yayue Dai, Jiangrong Yang, Wangsong Chen, Yucong Miao, Nenghui Pan, Zhenhua Li, Mingfei Shao

**Affiliations:** State Key Laboratory of Chemical Resource Engineering, College of Chemistry, Beijing University of Chemical Technology, Beijing 100029, China; Tianjin Key Laboratory of Brine Chemical Engineering and Resource Eco-utilization, College of Chemical Engineering and Materials Science, Tianjin University of Science and Technology, Tianjin 300457, China; State Key Laboratory of Chemical Resource Engineering, College of Chemistry, Beijing University of Chemical Technology, Beijing 100029, China; State Key Laboratory of Chemical Resource Engineering, College of Chemistry, Beijing University of Chemical Technology, Beijing 100029, China; State Key Laboratory of Chemical Resource Engineering, College of Chemistry, Beijing University of Chemical Technology, Beijing 100029, China; State Key Laboratory of Chemical Resource Engineering, College of Chemistry, Beijing University of Chemical Technology, Beijing 100029, China; State Key Laboratory of Chemical Resource Engineering, College of Chemistry, Beijing University of Chemical Technology, Beijing 100029, China; State Key Laboratory of Chemical Resource Engineering, College of Chemistry, Beijing University of Chemical Technology, Beijing 100029, China; State Key Laboratory of Chemical Resource Engineering, College of Chemistry, Beijing University of Chemical Technology, Beijing 100029, China

**Keywords:** photoelectrochemical, open-loop flow battery, cyclohexanone oxidation, adipic acid, adsorbed hydroxyl radicals

## Abstract

Photoelectrochemical (PEC) oxidation of cyclohexanone (CYC) to adipic acid offers a new sustainable synthesis approach, while efficient and selective transformation continues to pose a significant challenge. Moreover, traditional PEC systems typically require both sunlight and an external bias to ensure efficient and stable chemicals production, resulting in low energy utilization efficiency and 1-fold application scenarios. Herein, we construct a high-performance photoanode for PEC synthesis of adipic acid from CYC by modifying TiO_2_ nanorods array with NiFeCu (oxy)hydroxides (NiFeCu(OH)_2_/TiO_2_), achieving 5.6 μmol cm^−2^ h^−1^ adipic acid production with 95.7% selectivity at 1.0 V vs. reversible hydrogen electrode (RHE), outperforming the selectivity of the reported works for PEC CYC oxidation to adipic acid at low applied potentials. We demonstrate that the Cu modification in NiFe(OH)_2_/TiO_2_ promotes the generation of adsorbed hydroxyl radicals (OH*) to form reactive Ni/Fe^2+^*^δ^*−OH* species, facilitating the activation of C*_α_*−H bonds in CYC and the subsequent C−C cleavage to obtain adipic acid. It is notable that we achieved PEC CYC oxidation with redox flow batteries (denoted as PEC open-loop flow battery), in which value-added adipic acid is produced and electricity is discharged spontaneously (at a voltage of ∼1.3 V with a capacity of 1.1 Ah L^‒1^). This work underscores the potential for sustainable light-driven adipic acid synthesis and solar energy storage.

## Teaser

A novel photoelectrochemical (PEC) open-loop flow battery system, which integrates PEC CYC oxidation with the reduction of the vanadium pair (VO2^+^/VO2^+^), was designed to achieve adipic acid production coupled with energy storage.

## INTRODUCTION

The oxidative transformation of KA oil (a mixture of cyclohexanone (CYC) and cyclohexanol) into high-value adipic acid is a crucial reaction process in the polymer industry, with the resulting adipic acid product mainly used for Nylon-6,6 and fiber production [1–3]. In current industrial processes, elevated temperatures and 50–60 vol% nitric acid are typically required to drive and catalyze the reaction, which suffers from environmental issues [4,5]. Alternatively, hydrogen peroxide (H_2_O_2_) has been utilized as a green oxidant to synthesize adipic acid; however, the high cost and complex catalysts hinder scaled-up production [6,7]. In this scenario, a shift from traditional thermo-catalysis to efficient, environmentally friendly, and highly selective adipic acid production strategies ideally powered by renewable energy sources (for example, solar, wind, and hydro) and operated under mild conditions is highly desirable [8–12].

**Figure 1.**
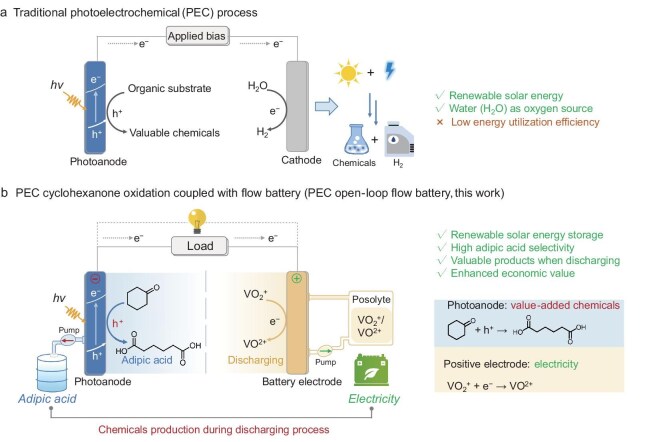
Schematic illustration of adipic acid synthesis. (a) Traditional PEC process. (b) Proposed PEC OLFB system for adipic acid production and electricity generation.

Recently, photoelectrochemical (PEC) techniques have emerged as an attractive approach to achieve direct solar-to-chemical conversion and greatly reduce the consumption of electric energy compared with the pure electrochemical methods, thus addressing the challenge of solar energy storage and meeting the demand for the utilization of mobile energy [13–17]. Specifically, PEC process can utilize photogenerated holes (h^+^) to generate active oxygen species or high-valence species *in situ* for oxidation processes, while cathodically producing hydrogen as a green fuel (Fig. 1a). Notably, adsorbed reactive oxygen species (for example, hydroxyl species OH*) can be formed *in situ* on the surface of semiconductor materials during water splitting [18–20], which are reactive and facilitate the dehydrogenation of C−H bond and cleavage of C−C bond in CYC molecules. Nevertheless, the insufficient amount of OH* species and the limited coverage of CYC substrates hinder C−C bond cleavage kinetics. Transition metal (for example, Ni, Co, and Fe) oxyhydroxide-based catalysts seemed to be an outstanding candidate for boosting the generation of high-valence metal-OH* active phases by facilitating charge carrier transport and increasing the number of surface-active sites [21,22]. For instance, doping Cu/V into Ni(OH)_2_ to tune the electronic properties of the targeted electrocatalyst can promote the formation of the Ni^3+^−OH* active phase and enhance CYC adsorption, thereby optimizing the performance in adipic acid production [5,9]. In this regard, we postulate that the precise design of oxyphilic active sites on the semiconductor may serve as OH* regulators and strengthen the adsorption of CYC, kinetically modifying the oxidation process.

Traditional PEC systems typically require both sunlight and an external bias to ensure efficient and stable chemicals production, resulting in low energy utilization efficiency and 1-fold application scenarios [23,24]. In this scenario, we are considering whether it is possible to design a new coupled reaction process by integrating PEC technology with energy storage systems, such as redox flow batteries (RFBs) [25,26]. The charging–discharging function of a conventional RFB is relying on the coupling of redox pairs in posolyte and negolyte [27]. The energy density (*E* = *Q* × Δ*U*) and power density (*P* = *I* × Δ*U*) requirements for a battery are satisfied by the potential difference (Δ*U*) between the redox pairs. Taking vanadium RFBs as an example, the redox potential difference between V^2^⁺/V^3^⁺ pair in negolyte (−0.26 V vs. standard hydrogen electrode (SHE)) and VO_2_⁺/VO^2^⁺ pair in posolyte (1.00 V vs. SHE) delivers an open circuit voltage (OCV) value of 1.26 V. Selecting couples in the negolyte with more negative equilibrium potentials than the V^2^⁺/V^3^⁺ pair can broaden the OCV of the batteries. Encouragingly, the oxidation potential of PEC organic molecules is determined by the position of the valence band edge [16,28–30], which enables the reaction to occur at a lower potential, such as for CYC (−0.64 V vs. SHE, under illumination, in 0.5 M KOH) [8]. In this context, we hypothesize that replacing the conventional redox reaction at the negative electrode (for example, V^3^⁺/V^2^⁺) with PEC selective oxidation of organic molecules could enable a flow battery system to expand the OCV and produce high-value-added chemicals (for example, adipic acid) during discharge. We denote this novel system as PEC open-loop flow battery (OLFB) (Fig. 1b), distinguishing it from conventional light-assisted RFBs, where the electrolyte circulates in a closed-loop manner.

Herein, we designed a TiO_2_-supported NiFeCu (oxy)hydroxide (NiFeCu(OH)_2_/TiO_2_) as an efficient photoanode for CYC oxidation, achieving an adipic acid production rate of 5.6 μmol cm^−2^ h^−1^ with 95.7% selectivity at 1.0 V vs. reversible hydrogen electrode (RHE) in the H-type quartz cell. To improve the efficiency of solar energy storage and conversion, the PEC CYC oxidation was integrated with the reduction of vanadium pair (VO_2_⁺/VO^2^⁺) to construct a new PEC OLFB system. As expected, the solar-driven OLFB discharged at ∼1.3 V, delivering a capacity of 1.1 Ah L⁻^1^ and simultaneously producing adipic acid with a selectivity of ∼94%. Experimental and theoretical evidence revealed that Ni/Fe^2+^*^δ^*−OH* species serve as the actual reactive phase for CYC oxidation under illumination, which is *in situ* generated from the oxidation of NiFe(OH)_2_ by the photogenerated holes. Furthermore, the Cu incorporation not only promotes the generation of Ni/Fe^2+^*^δ^*−OH* species, but also enhances the adsorption and conversion of CYC, thus boosting adipic acid formation. This PEC OLFB system not only achieves efficient utilization and conversion of solar energy, but also advances the PEC application scenarios.

## RESULTS AND DISCUSSION

### Photoelectrosynthesis of adipic acid

**Figure 2.**
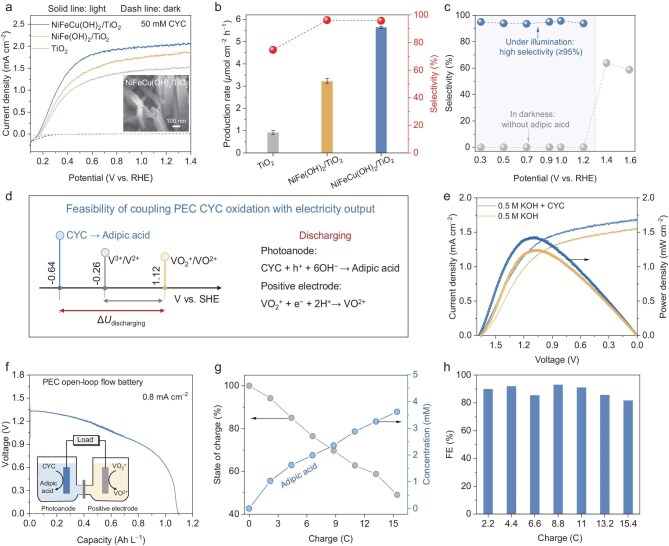
Photoelectrosynthesis of adipic acid and discharge feasibility of PEC OLFB. (a) LSV curves of NiFeCu(OH)_2_/TiO_2_, NiFe(OH)_2_/TiO_2_, and pristine TiO_2_ photoanodes at a scan rate of 10 mV s^−1^ in 0.5 M KOH with 50 mM CYC under AM 1.5 G (100 mW cm^−2^) illumination and dark conditions. Inset: Top view SEM image of NiFeCu(OH)_2_/TiO_2_ photoanode. Scale bar: 100 nm. (b) Production rate and selectivity of adipic acid over NiFeCu(OH)_2_/TiO_2_, NiFe(OH)_2_/TiO_2_, and pristine TiO_2_ photoanodes. Reaction conditions: 0.5 M KOH electrolyte with 50 mM CYC at 1.0 V vs. RHE for 2 h under illumination. Note: the error bars (standard deviation) were obtained by three parallel repeated results, and the corresponding data represent the average values. (c) Selectivity of adipic acid at different potentials (0.3–1.6 V vs. RHE) over NiFeCu(OH)_2_/TiO_2_ photoanode under illumination or dark conditions. (d) The oxidation potentials of CYC, potentials of the redox couples in traditional RFB, and chemical equations for the PEC CYC oxidation coupled with flow battery. (e) Polarization and power density curves of the PEC OLFB with/without CYC in 0.5 M KOH as the negolyte in the H-type cell. (f) Discharge curve of the PEC OLFB with 50 mM CYC in 0.5 M KOH as the negolyte with a current density of 0.8 mA cm^−2^. The inset shows the corresponding scheme of the redox reactions during its discharge process. (g) Time-dependent SOC, concentration of adipic acid product, and (h) total product Faradaic efficiency during the discharge process of the PEC OLFB.

In previous reports, high-valence metal-OH* have been indicated critical for CYC selective oxidation and the dehydrogenation of carbonaceous intermediates [31–34]. In addition, transition metal oxyhydroxide-based catalysts show great potential for enhancing the generation of high-valence metal-OH* active phase [35–37]. Inspired by this knowledge, we thought to explore a photoanode design by modifying NiFeCu(OH)_2_ on traditional TiO_2_ sample (denoted as NiFeCu(OH)_2_/TiO_2_). For comparation, NiFe(OH)_2_-decorated TiO_2_ (NiFe(OH)_2_/TiO_2_) was also prepared through the same electrochemical deposition process (see experimental section). Scanning electron microscopy (SEM) image displayed that the amorphous NiFeCu(OH)_2_ is uniformly distributed on the surface of TiO_2_ nanorod arrays (Fig. 2a and Fig. S1). Moreover, the atomic ratio of Ni/Fe/Cu in NiFeCu(OH)_2_ is close to 1.0/4.0/0.7, and the content of Cu is detected by inductively coupled plasma-optical emission spectrometer (ICP-OES) (Table S1). More detailed structural characterizations are provided in supplementary information (Figs S2–S11 and Note S1).

The as-prepared NiFeCu(OH)_2_/TiO_2_ photoanodes were employed for the selective oxidation of CYC. For the PEC oxidation of CYC in an aqueous medium, water oxidation can be the major competing reaction. Therefore, the PEC oxidation of water and CYC was first investigated in a 0.5 M KOH electrolyte under light illumination (AM 1.5 G, 100 mW cm^–2^) by linear sweep voltammetry (LSV). All photoanodes show no anodic photocurrent response in the dark throughout the test region (0.4–1.4 V vs. RHE), indicating the necessity of light illumination (Fig. 2a). The pristine TiO_2_ exhibits a photocurrent density of 1.35 mA cm^–2^ at 1.0 V vs. RHE for water oxidation. The introduction of (oxy)hydroxide enhances photocurrent densities of both NiFe(OH)_2_/TiO_2_ and NiFeCu(OH)_2_/TiO_2_ photoanodes (Fig. S12). To understand the promoted photocurrent densities of photoanode for CYC oxidation, we evaluated the optical properties, charge separation and transfer kinetics of different photoanodes. The enhanced charge injection efficiency, extended average charge lifetime, and other tests (details are discussed in Figs S13–S19 and Note S2) suggest that NiFeCu(OH)_2_ modification can inhibit the photogenerated carrier recombination, while simultaneously promote carrier separation and transfer thus leading to a higher photocurrent.

When 50 mM CYC was added, the anodic current densities increased for all photoanodes, particularly for the NiFeCu(OH)_2_/TiO_2_ photoanode (1.98 mA cm^–2^ at 1.0 V vs. RHE), suggesting that the CYC oxidation is thermodynamically more favorable than water oxidation as the anode reaction. Notably, after adding CYC, the photocurrent density of NiFeCu(OH)_2_/TiO_2_ increased by 0.31 mA cm^–2^, which is 4.4 and 1.3-fold higher compared to pristine TiO_2_ and NiFe(OH)_2_/TiO_2_ (0.07 and 0.24 mA cm^–2^), respectively (Fig. 2a). These results demonstrate the positive effect of NiFeCu(OH)_2_ modification on PEC CYC oxidation.

We then evaluated the PEC CYC oxidation performance at a constant potential of 1.0 V vs. RHE in 0.5 M KOH containing 50 mM CYC under light illumination. The oxidation products were identified and quantified using high-performance liquid chromatography (HPLC). We found that adipic acid was the main product of CYC oxidation, with glutaric acid as the by-product over the prepared photoanodes (Fig. S20). Figure 2b illustrates the production rate and selectivity of adipic acid from PEC CYC oxidation over different photoanodes and the corresponding Faradaic efficiencies (FE) are displayed in Fig. S21. The NiFeCu(OH)_2_/TiO_2_ photoanode achieves the highest adipic acid production rate of 5.6 μmol cm^‒2^ h^‒1^ at 1.0 V vs. RHE, with a high selectivity of 95.7% and FE reaching 82.6%. This represents a 5.8-fold improvement compared to the pristine TiO_2_ photoanode (0.9 μmol cm^–2^ h^–1^, 74.6% selectivity, and 28.6% FE). In comparison, the NiFe(OH)_2_/TiO_2_ photoanode exhibits inferior PEC performance in selective CYC oxidation with a production rate of 3.2 μmol cm^–2^ h^–1^, a selectivity of 96.1%, and an FE of 62.3%, suggesting that the incorporation of NiFeCu(OH)_2_ promotes the PEC CYC oxidation process. Overall, the selectivity of adipic acid over the developed NiFeCu(OH)_2_/TiO_2_ photoanode outperforms all the previously reported PEC works for synthesizing adipic acid (Table S2).

We further explored the influence of applied potentials on the product distribution using NiFeCu(OH)_2_/TiO_2_ photoanode (Fig. 2c and Fig. S22). It was observed that CYC can be converted to adipic acid at an ultra-low potential of 0.3 V vs. RHE. As the applied potential increased from 0.3 V vs. RHE to 1.0 V vs. RHE, the production rate of adipic acid gradually increased from 2.6 to 5.6 μmol cm^‒2^ h^‒1^, while the selectivity of adipic acid remained almost consistent (≥94%) and the carbon efficiency reached ∼98% (Fig. S23). This highlights the efficacy of the PEC approach for adipic acid synthesis over a wide potential window. Further increasing the potential to 1.2 V vs. RHE does not enhance adipic acid production but reduces adipic acid selectivity (83.0%) due to the occurrence of undesirable side reactions (for example, undesired glutaric acid by-product). When the reactions were performed in the dark (electrocatalysis), CYC could not be converted to adipic acid until a potential above 1.4 V vs. RHE, resulting in poor adipic acid generation and selectivity (0.5 μmol cm^–2^ h^–1^, ∼60.0%) (Fig. 2c). Furthermore, the production of adipic acid was significantly suppressed only under AM 1.5 G illumination (photocatalysis), indicating the unique advantages of PEC system in selective oxidation of CYC under mild conditions (Fig. S24). The solar-to-adipic acid quantum efficiency and photon-to-current conversion efficiencies (IPCE) achieve 56.0% and 69.4% at a wavelength of 380 nm over NiFeCu(OH)_2_/TiO_2_ photoanode (Fig. S25). The production rate and selectivity of adipic acid over NiFeCu(OH)_2_/TiO_2_ photoanode can be maintained after 20 batches (40 h) (detailed in Figs S26 and S27, and Note S3). Moreover, Cu^2+^ leaching into the electrolyte was negligible, amounting to only ∼0.21% after 20 h and 0.29% after 40 h, indicating that the NiFeCu(OH)_2_/TiO_2_ photoanode remains stable in our PEC reaction conditions (Fig. S27, Table S3, and Note S4).

### Photoelectrosynthesis of adipic acid coupled with discharge in H-type cell

The above results show that adipic acid product can be obtained via a PEC method; however, challenges remain in achieving higher solar conversion efficiency, as the traditional PEC systems still need the input of solar energy and external electrical energy [38–40]. Coupling PEC cells with a RFB enables simultaneous discharging and value-added chemical production, which can achieve both high-efficiency solar energy storage and conversion. Therefore, we sought to establish a PEC OLFB system for the simultaneous production of electricity and adipic acid by combining VO_2_⁺ reduction in the posolyte with CYC oxidation in the negolyte, aiming to obtain higher solar conversion efficiency.

We first investigated the feasibility of pairing the VO_2_^+^/VO^2+^ redox couple with CYC oxidation in the PEC OLFB system. By employing LSV and cyclic voltammetry tests in a three-electrode cell, the OCV values and the discharging feasibility of PEC OLFB were assessed. As shown in Fig. S29 and Fig. 2d, the onset potential for the CYC oxidation reactions under illumination is −0.64 V vs. SHE, which is much lower than that of the V^3+^/V^2+^ redox couple (−0.26 V vs. SHE). Note that VO_2_^+^/VO^2+^ redox shows the positive potential of 1.12 V vs. SHE, implying a relatively larger discharge voltage in the PEC OLFB. Therefore, when the CYC-to-adipic acid conversion is paired with the VO_2_^+^/VO^2+^ positive redox couple, it leads to a theoretical OCV of 1.76 V and consequently enhancing the energy density and power density of battery (details in Note S5).

The PEC tests were then conducted using a two-electrode setup in an H-type cell, with full-spectrum light illumination (AM 1.5 G, 100 mW cm^−2^) to assess the feasibility of coupling CYC oxidation with electricity generation. As illustrated in Fig. S30, a two-electrode PEC OLFB is constructed from a NiFeCu(OH)_2_/TiO_2_ photoanode immersed in an aqueous solution of 50 mM CYC and 0.5 M KOH, and a graphite felt counter electrode immersed in an aqueous solution of VO_2_^+^ and 2 M H_2_SO_4_, separated by a cation conductive Nafion-117 membrane. The VO_2_^+^/VO^2+^ redox pair of all-vanadium RFB was selected as the posolyte due to its maturity, and the state of charge (SOC) was maintained at 100% through charging for the subsequent discharge testing (detailed information about the charging process is provided in Fig. S31 and Note S6).

The polarization and power density curves were measured under illumination to evaluate the power generation performance of the PEC OLFB. As shown in Fig. 2e, the PEC OLFB exhibited an OCV of 1.6 V, a short-circuit current of 1.5 mA cm^−2^, and a maximum power density of 1.2 mW cm^−2^ in 0.5 M KOH using NiFeCu(OH)_2_/TiO_2_. When CYC was employed as the organic substrate, a higher short-circuit current of 1.7 mA cm^−2^ and power density of 1.4 mW cm^−2^ were realized due to the kinetically favorable CYC oxidation reaction. Furthermore, this system exhibited an outstanding PEC stability for electricity generation under consecutive on–off light illumination (Fig. S32). The PEC OLFB can be discharged at a voltage of ∼1.3 V with a capacity of 1.1 Ah L^–1^ when applying a discharge current of 0.8 mA cm^–2^ (Fig. 2f). The oxidation products were then analyzed using HPLC, which revealed adipic acid as the main product with a selectivity of 94.5% (Fig. S33). After the battery is nearly fully discharged, a selectivity of 92% and a FE of 81.7% for adipic acid were achieved, and the SOC reached 60% (Fig. 2g and h, the SOC of the VO_2_^+^/VO^2+^ anodic electrolyte was assessed with an ultraviolet-visible spectrophotometer). The PEC OLFB developed in this work can simultaneously achieve adipic acid production and renewable electricity storage, highlighting its potential for the efficient conversion of solar energy into chemical and electric energy.

### Photoelectrosynthesis of adipic acid coupled with discharge in flow cell

**Figure 3.**
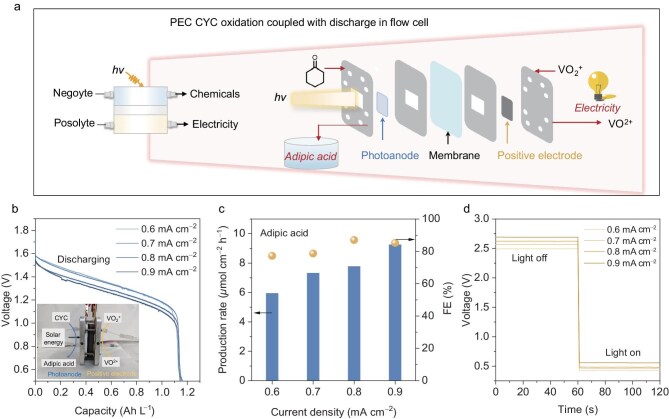
Performances of PEC OLFB in the flow cell. (a) Schematic diagram of the PEC OLFB system. (b) Discharge performance of the PEC OLFB at different current densities. Inset: The photograph of the PEC OLFB cell stack and the redox reactions during its discharge process. (c) Production rate and FE of adipic acid after the battery discharge at different current densities. (d) Charging potential of PEC OLFB under different current densities with/without illumination.

In light of the excellent PEC performance of NiFeCu(OH)_2_/TiO_2_ photoanode and the discharge property in H-type cell, we further constructed a prototype reaction device of PEC OLFB for the pursuit of continuous production of adipic acid and electricity generation. The cell stack and system of the reversible PEC OLFB was given in Fig. 3a. In this flow cell, the illumination area of NiFeCu(OH)_2_/TiO_2_ photoanode is 9 cm^2^ (a 3 cm × 3 cm flow cell), and a graphite felt was used as the negative electrode. To evaluate the power generation performance of PEC OLFB with CYC used as the negolyte, the voltage–current curves were measured. As shown in Fig. S34, the battery exhibited an OCV of 1.7 V, a maximum power density of 1.7 mW cm^−2^, and a short-circuit current of 1.8 mA cm^−2^. We then explore the potential of the PEC OLFB for the continuous synthesis of adipic acid and its discharging capabilities. As depicted in Fig. 3b, the battery maintained a discharge voltage of ∼1.5 V and discharge capacity are stable at 1.1 Ah L^−1^ at a current density ranging from 0.6 to 0.9 mA cm^−2^, achieving a smoothly discharge process. The adipic acid production rate showed a positive correlation with current density, increasing from 6.0 to 9.3 μmol cm^−2^ h^−1^ over the range of 0.6–0.9 mA cm^−2^ and maintaining FE and selectivity above 77% (Fig. 3c). Furthermore, the solar-to-chemical efficiency (*η*_STC_) was calculated by normalizing the output power density to the incident solar irradiance, while the energy efficiency (EE) was defined as the ratio of discharge to charge energy (see the relevant formula in supplementary information). As shown in Fig. S35, the *η*_STC_ of PEC OLFB gradually increased from 2.1% to 2.7% as the voltage rose from 0.3 to 0.9 V. Further increasing the voltage to 1.3 V did not enhance *η*_STC_. Meanwhile, the EE ranges from 58.7% at 0.6 mA cm^−2^ to 40.8% at 0.9 mA cm^−2^ current densities.

Moreover, the conventional negative redox couple in the RFB was replaced by the HER during charging. We validated the feasibility of the battery charging process. As depicted in Fig. 3d and Fig. S36, in the absence of light, the battery required a charging voltage of 2.69 V at a current density of 0.9 mA cm^−2^. Even at a lower current density of 0.6 mA cm^−2^, a charging voltage of 2.49 V was still needed. It is worth noting that under illumination, the charging voltage decreased significantly by 2 V (from 2.49 to 0.43 V at a current density 0.6 mA cm^−2^), indicating the positive effect of solar energy input on the flow battery. Notably, the PEC OLFB system allows convenient and low-cost scaling of energy storage capacity by simply increasing the volume of liquid electrolytes in the storage tanks. In this configuration, the energy storage capacity is limited only by the tank size, and the cost per unit of stored energy decreases significantly as the system is scaled up [41]. These results suggest the potential for directly converting intermittent solar energy into chemical energy and electrical energy within this coupling system, offering a more environmentally friendly and energy-efficient approach.

In summary, during charging, the PEC OLFB pair the oxidation of VO^2^⁺ to VO_2_⁺ with HER, while discharging involves oxidation of CYC to adipic acid with VO_2_⁺ reduction to VO^2^⁺. This open-loop mechanism fundamentally distinguishes this system from conventional light-assisted RFB, in which electrolytes circulate in a closed loop without chemical synthesis. Specifically, under illumination, photogenerated charge carriers (holes or electrons) drive the oxidation or reduction of redox couples within the electrolytes, thereby generating the charged species in the RFB. During the subsequent discharge phase, these species undergo reversible redox reactions to release energy [25,26]. For PEC hybrid systems coupled with organic oxidation, solar energy is stored as chemical energy through irreversible reaction pathways [42,43]. Although the co-production of value-added chemicals offers significant economic potential, the thermodynamic irreversibility of these reactions restricts such systems to primary battery-like operation, rendering them unsuitable for rechargeable energy storage applications (Fig. S37 and Table S4).

### Mechanism study

**Figure 4.**
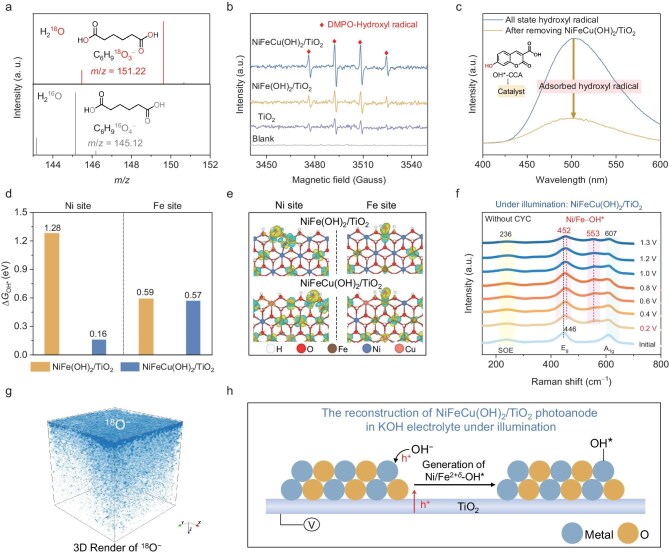
PEC properties of NiFeCu(OH)_2_/TiO_2_. (a) LC-MS spectra of the adipic acid product using isotope-labeled electrolyte with 10% H_2_^18^O. (b) EPR spectra of TiO_2_, NiFeCu(OH)_2_/TiO_2_ and NiFe(OH)_2_/TiO_2_ photoanodes in 0.5 M KOH under AM 1.5 G (100 mW cm^−2^) illumination using DMPO as a spin-trapping agent. (c) Fluorescence spectra for the detected adsorbed OH* using CCA indicators. CCA can capture reactive hydroxyl species to form CCA-OH; when the photoanode was removed, fluorescence intensity decreased significantly. (d) Gibbs free energy of OH* formation over NiFe(OH)_2_/TiO_2_ and NiFeCu(OH)_2_/TiO_2_. (e) Charge density difference for OH* adsorption on Ni and Fe sites for NiFe(OH)_2_/TiO_2_ and NiFeCu(OH)_2_/TiO_2_. Iso-values are 0.003 eV/Å^3^ for charge density. (f) *In-situ* Raman spectra of NiFeCu(OH)_2_/TiO_2_ photoanode in 0.5 M KOH under illumination. (g) TOF-SIMS 3D imaging of ^18^O on the surface of the NiFeCu(OH)_2_/TiO_2_ photoanode. (h) Schematic illustration of the formation of Ni/Fe^2+^*^δ^*−OH* in NiFeCu(OH)_2_/TiO_2_ photoanode.

It is noted that the photocurrent densities of NiFe(OH)_2_/TiO_2_ and NiFeCu(OH)_2_/TiO_2_ were comparable (1.4 vs 1.6 mA cm^‒2^ at 1.0 V vs. RHE), whereas the production rate of adipic acid on NiFeCu(OH)_2_/TiO_2_ was ∼2 times higher than that on NiFe(OH)_2_/TiO_2_ (Fig. S12 and Fig. 2a). To further understand the promoting effect of NiFeCu(OH)_2_ modification on adipic acid production, we diverted our attention to mechanistic studies. The oxygen source of adipic acid product was firstly identified using a 0.5 M KOH solution containing 10% H_2_^18^O as the electrolyte. The liquid chromatography-mass spectrometry (LC-MS) results in Fig. 4a show that ^18^O can be detected in adipic acid in the form of C_6_H_9_O^18^O_3_^−^ (*m/z* = 151.22), indicating that H_2_O serves as the main oxygen source for the PEC CYC oxidation to synthesize adipic acid. *In situ* electron paramagnetic resonance (EPR) measurement using 5,5-dimethyl-1-pyrroline *N*-oxide (DMPO) serving as a trapping agent was then conducted to capture active intermediates adsorbed on the catalyst surface [44]. As shown in Fig. 4b, only hydroxyl radical signals generated from OH^−^ or H_2_O oxidation by photogenerated holes were detected in the absence of CYC. Moreover, NiFeCu(OH)_2_/TiO_2_ exhibited a higher intensity of the DMPO-OH adduct compared to NiFe(OH)_2_/TiO_2_ and pristine TiO_2_ photoanodes under light illumination, indicating that the introduction of NiFeCu(OH)_2_ can boost the generation of hydroxyl radical species. Correspondingly, fluorescence experiments using coumarin-3-carboxylic acid (CCA) as the probe were conducted to elucidate the existing state of hydroxyl radical species in this system (the detailed principle is provided in Fig. S38). CCA probe can capture the hydroxyl radicals to form CCA-OH, which then adsorb on the surface of catalysts [45]. After removing NiFeCu(OH)_2_/TiO_2_, the fluorescence intensity of CCA-OH at ∼503 nm decreased significantly, indicating that hydroxyl radicals are mainly adsorbed on the surface of NiFeCu(OH)_2_/TiO_2_ (denoted as adsorbed OH*) (Fig. 4c and Fig. S39).

Furthermore, we calculated the Gibbs free energy of OH* formation (Δ*G*_OH*_) for the NiFe(OH)_2_/TiO_2_ and NiFeCu(OH)_2_/TiO_2_ photoanodes. As shown in Fig. 4d and Fig. S40, the formation energy of OH* at Ni sites over NiFeCu(OH)_2_/TiO_2_ (0.16 eV) is significantly lower than that in NiFe(OH)_2_/TiO_2_ (1.28 eV), indicating that Cu incorporation facilitates OH* formation at Ni centers. Charge density difference calculations demonstrate that Cu doping triggers strong electronic polarization at Ni sites during OH* adsorption (Fig. 4e and Fig. S41), thereby promoting photogenerated hole trapping and enhancing OH* adsorption. Density of states analysis (Fig. S42) indicates that the *d*-band center of Ni sites in NiFeCu(OH)_2_/TiO_2_ is closer to the Fermi energy level than that of NiFe(OH)_2_/TiO_2_, suggesting that Ni sites in NiFeCu(OH)_2_/TiO_2_ feature less anti-bonding states, which enables interaction between Ni sites and OH* (details in Note S7). Upon identification of the reactive species, a series of quenching experiments were conducted to confirm the contribution of different reactive species to PEC CYC oxidation (Fig. S43 and Note S8). The results show that the reaction was significantly inhibited after the addition of Na_2_SO_3_ (h^+^ scavenger) and *tert*-butanol (TBA, OH* scavenger), implying that the OH* species which are generated through the oxidation of OH^−^ by h^+^ are the key species involved in the CYC oxidation for generating adipic acid.

According to previous works, during PEC oxidation of alcohols and other nucleophilic substrates catalyzed by transition metal oxyhydroxides-based catalysts, they first lose electrons and then undergo reconstruction into high-valence metal-OH* species [46–49]. Then, the high-valence metal-OH* (for example, Ni^3+^−OH*, Ni^2+^*^δ^*−OH*, and Co^3+^−OH*) serve as the authentic active species for CYC oxidation and C−C bond cleavage by abstracting hydrogen and electron from the nucleophilic substrates [9]. Therefore, we first demonstrated that Ni/Fe^2+^*^δ^*−OH* serves as the active phase for CYC oxidation over both NiFeCu(OH)_2_/TiO_2_ and NiFe(OH)_2_/TiO_2_ using *in situ* Raman technique, by observing Ni/Fe^2+^*^δ^*−OH* formation at 452 and 553 cm^‒1^ in the 0.2–1.3 V vs. RHE potential window and its subsequent consumption after introducing CYC (Fig. 4f and Figs S44–S46). This is also confirmed by multi-potential step chronoamperometric measurement (Fig. S47). After adding 50 mM CYC into the system, the reduction current disappeared under an open circuit conditions, indicating that the Ni/Fe^2+^*^δ^*−OH* is consumed by CYC via a spontaneous dehydrogenation process. This result further proves that the surface Ni/Fe^2+^*^δ^*−OH* species serve as the authentic active species for CYC oxidation. The reactivity of the reconstructed NiFeCu(OH)_2_ surpasses that of NiFe(OH)_2_, as evidenced by the enhanced Raman signal for Ni/Fe^2+^*^δ^*−OH*, and NiFeCu(OH)_2_/TiO_2_ exhibits a lower positive potential for Ni/Fe^2+^*^δ^*−OH* generation compared with NiFe(OH)_2_/TiO_2_ under the conditions without CYC (Fig. S48). These results suggest that the superior performance of NiFeCu(OH)_2_/TiO_2_ in CYC oxidation may be due to the Cu modification, which promotes the formation of Ni/Fe^2+^*^δ^*−OH* active phase.

To probe the evolution of the Ni/Fe^2+^*^δ^*−OH* in the reconstruction process of catalyst, time-of-flight secondary ion mass spectrometry (TOF-SIMS) experiments were employed using NiFeCu(OH)_2_/TiO_2_ catalyst in 0.5 M KOH solution containing 10% H_2_^18^O at 1.0 V vs. RHE for 600 s. As shown in Fig. 4g and Fig. S49, the presence of ^18^O from electrolyte was detected in the outermost layer of the catalyst with a blue decay gradient, indicating a gradual decrease of ^18^O. It can be concluded that the oxygen source of Ni/Fe^2+^*^δ^*−OH* originates from the adsorbed OH* generated by OH^−^/H_2_O. Collectively, we propose a reconstruction mechanism from the NiFeCu(OH)_2_ phase to a high-valence metal-OH* phase (Fig. 4h and Fig. S50). First, the surface of NiFeCu(OH)_2_ undergoes dehydrogenation reconstruction in an alkaline electrolyte, resulting in an increase in the valence of Ni/Fe and the deprotonation of hydroxyl groups, which exposes surface active oxygen sites. Next, the negatively charged active oxygen induces the rapid migration of h^+^ to the surface catalysts. The captured holes break the Ni/Fe–O bond *in situ* to create active sites. Subsequently, OH* from the oxidation of OH^−^ by h^+^ adsorbs onto the photoanode surface to form Ni/Fe^2+^*^δ^*−OH* active phase.

**Figure 5.**
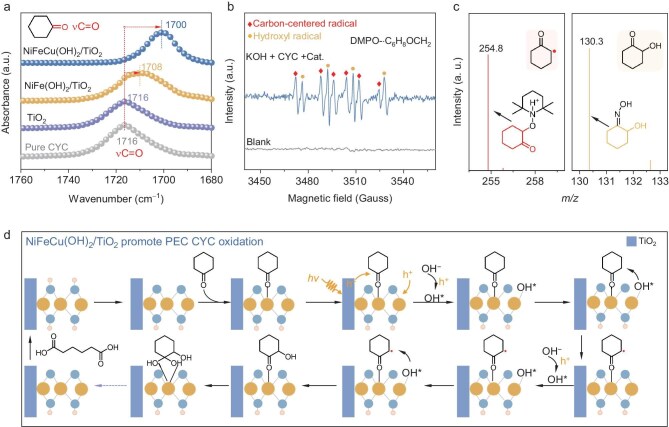
The investigation of PEC CYC oxidation process. (a) Diffuse reflectance infrared Fourier transformation spectroscopy of CYC adsorbed on TiO_2_, NiFeCu(OH)_2_/TiO_2_, and NiFeCu(OH)_2_/TiO_2_ photoanodes. (b) EPR spectrum of NiFeCu(OH)_2_/TiO_2_ in 0.5 M KOH containing 50 mM CYC. (c) LC-MS mass spectra of TEMPO-C (left) and the condensation product (right). (d) Schematic illustration of PEC CYC oxidation to adipic acid over NiFeCu(OH)_2_/TiO_2_ photoanode.

The above analysis demonstrates that Cu modification induces a rapid phase reconstruction of NiFeCu(OH)_2_, which enhances the rate of the CYC oxidation reaction. However, Ni/Fe^2+^*^δ^*−OH* sites can also promote OER activity, as evidenced by the increased photocurrent density observed for NiFeCu(OH)_2_/TiO_2_ compared to NiFe(OH)_2_/TiO_2_ in the absence of CYC (Fig. S12). Therefore, the superior performance of adipic acid over NiFeCu(OH)_2_/TiO_2_ cannot be solely attributed to the enhanced phase reconstruction induced by Cu modification. It is widely known that the adsorption behavior of reactants can regulate the coverage of reactant over the catalyst surface [20,50], thus the FE and production rate of the desired product can be improved (Fig. S51). As shown in Fig. 5a, the diffuse reflectance infrared fourier transformation (DRIFT) spectrum of gaseous CYC displays a characteristic peak at 1716 cm^−1^, corresponding to the stretching vibration of the carbonyl (*ν*(C=O)) bond in CYC [51,52]. When CYC was introduced with Ar as the carrier gas and subsequently purged physically adsorbed CYC molecules, the *ν*(C=O) band both showed red-shifts due to the adsorption of CYC over the surface of NiFe(OH)_2_/TiO_2_ and NiFe(OH)_2_/TiO_2_. Notably, the *ν*(C=O) band signal of NiFeCu(OH)_2_/TiO_2_ shifted to a much lower wavenumber (1700 cm^−1^) compared to that of NiFe(OH)_2_/TiO_2_, indicating stronger CYC adsorption over NiFeCu(OH)_2_/TiO_2_. In contrast, the DRIFT spectrum of pristine TiO_2_ did not show significant changes, suggesting a relative weaker adsorption of CYC.

To further verify enhanced CYC adsorption over NiFeCu(OH)_2_/TiO_2_, we employed quartz crystal microbalance measurement to monitor the mass change of the adsorbed substrates in real-time. As shown in Fig. S52, the modification of NiFeCu(OH)_2_ exhibited an obvious increase in mass after CYC was added, indicating that the incorporation of Cu enhances the adsorption of the CYC substrate, which is consistent with the DRIFT spectrum results. The spin-polarized density functional theory calculations were further performed to investigate the adsorption behavior of CYC over designed photoanodes. CYC can be either adsorbed at Ni or Fe sites over NiFeCu(OH)_2_/TiO_2_ and NiFe(OH)_2_/TiO_2_ (Fig. S53). The calculated adsorption energies (*E*_ads_) reveal that Cu doping significantly enhances adsorption strength, particularly at the Fe site where the energy decreases from −0.29 to −1.37 eV, which is consistent with the observed stronger CYC adsorption over NiFeCu(OH)_2_/TiO_2_. This is also confirmed by open-circuit potential measurements (Fig. S54). Moreover, charge density difference and Bader charge calculations reveal a distinct preference for electron transfer during CYC adsorption at Fe sites compared to Ni sites, which is consistent with their surface electronic character in both photoanodes (Figs S55 and S56). Collectively, (oxy)hydroxides, paticularly the NiFeCu(OH)_2_ modification can enhance the adsorption capacity of C=O bond to enrich CYC molecules at Fe sites on the catalyst surface, which facilitates efficient adipic acid production.

In addition to investigating the impact of Cu modification on the generation of Ni/Fe^2+^*^δ^*−OH* species and CYC adsorption, we also evaluated whether Cu modification alters the oxidation route of CYC toward adipic acid formation. According to previous work, several possible intermediates were introduced into the PEC CYC oxidation reaction [5,8–10]. As shown in Figs S57 and S58, the results indicate that 2-hydroxycyclohexanone is a key intermediate in the selective oxidation of CYC to adipic acid. To verify this process, *in situ* EPR was conducted by employing DMPO for capturing active intermediates adsorbed on the catalyst surface. In Fig. 5b, both DMPO-OH and DMPO-C spin adducts are observed upon the addition of CYC over both catalysts. Notably, a decrease in OH* signals is observed after the addition of CYC, indicating that the crucial role of OH* species in the cleavage of C−H bonds in CYC. Then, a 2,2,6,6-tetramethyl-1-piperidinyloxy (TEMPO) radical scavenger was introduced into the reaction to further examine and analyze the C-centered radicals (Fig. S59) [53]. C-centered radical intermediates at the C*_α_* position (*m/z* value of 254.8) were detected by LC-MS (Fig. 5c, left). More experimental data are provided in Supporting Information (Figs S60 and S61, and Note S9). These results clearly evidenced the production of C*_α_*-centered radical intermediates during adipic acid generation.

To illustrate the possible downstream pathway for PEC CYC oxidation subsequent to the formation of C*_α_*-centered radicals, NH_2_OH was added into the reaction system (Fig. S62), and the *m*/*z* value of 130.3 assigned to 2-hydroxycyclohexanone oxime was detected, strongly confirming the existence of 2-hydroxycyclohexanone (Fig. 5c, right). These results clearly demonstrate that the formation of C*_α_*-centered radical intermediate occurs through the dehydrogenation of C*_α_*‒H of CYC, followed by the subsequent oxygenation of C*_α_*-centered radicals by OH* to yield 2-hydroxycyclohexanone. We further employed 2-hydroxycyclohexanone as the starting reactant under the same conditions to validate whether NiFeCu(OH)_2_ alters the subsequent oxidation step. The results in Fig. S63 showed comparable product selectivity, indicating that PEC CYC oxidation over NiFe(OH)_2_/TiO_2_ and NiFeCu(OH)_2_/TiO_2_ follows a similar route.

Specifically, the surface of NiFeCu(OH)_2_ undergoes spontaneous dehydrogenation reconstruction in the KOH electrolyte and the reaction starts with the spontaneous adsorption of C=O in CYC on the photoanode. Then Ni/Fe–O bond and OH^−^ are activated by photogenerated holes to *in situ* generate Ni/Fe^2+^*^δ^*−OH* active species, as confirmed by the *in situ* Raman spectra in Fig. 4f. The generated Ni/Fe^2+^*^δ^*−OH* reactive center robs H atom of CYC to form C*_α_*-centered radicals. Subsequently, OH* is regenerated through the oxidation of OH^−^ by the hole and reacts with the C*_α_*-centered radicals to form the 2-hydroxycyclohexanone intermediate. Then, the 2-hydroxycyclohexanone intermediate undergoes hydration and C*_α_*−C*_β_* bond cleavage processes, ultimately producing adipic acid. Finally, adipic acid desorbs from the surface of the photoanode and the surface of the photoelectrode is restored to the activated state to complete a cycle (Fig. 5d). Moreover, for adipic acid oxidation, the photocurrent density and conversion rate were much lower than those of CYC (Fig. S64), suggesting adipic acid is relatively stable and can be accumulated in our PEC reaction conditions. This stability may be attributed to the inability of the carboxyl groups to sufficiently activate adjacent C−H bonds, while the central methylene units retain a strong, alkane-like character resistant to mild cleavage. Additionally, the saturated C−C backbone is thermodynamically stable. Glutaric acid, observed as a byproduct during adipic acid formation over NiFeCu(OH)_2_/TiO_2_ photoanode (Fig. S20), is likely formed via a 3-hydroxycyclohexan-1-one intermediate during CYC oxidation [5,8,9,33], which undergoes hydration and C−C bond cleavage to produce glutaric acid (Fig. S65).

## CONCLUSION

In summary, we successfully achieved PEC CYC oxidation to adipic acid with high selectivity even at a ultra-low potential of 0.3 V vs. RHE by modifying NiFeCu(OH)_2_ on TiO_2_ photoanode. The adipic acid selectivity over NiFeCu(OH)_2_/TiO_2_ photoanode reaches ∼95% at a wide potential range from 0.3 to 1.3 V vs. RHE and a maximum production rate of 5.6 μmol cm^−2^ h^−1^. Furthermore, we demonstrated the feasibility of PEC OLFB system by combining the NiFeCu(OH)_2_ photoanode with a flow battery positive electrode (graphite felt in VO_2_^+^/VO^2+^ electrolyte), simultaneously achieving adipic acid production and renewable electricity storage. Experimental and calculation studies reveal that high-valence Ni/Fe^2+^*^δ^*‒OH* is the primary active species for CYC oxidation. The modification of NiFeCu(OH)_2_ can enhance CYC substrates adsorption and promote the generation of Ni/Fe^2+^*^δ^*‒OH*species, which facilitate the oxidation and C‒C bond cleavage of CYC to produce adipic acid. The charge kinetics analysis indicated (oxy)hydroxides, particularly NiFeCu(OH)_2_ modification can facilitate photogenerated carrier separation and transfer. This work provides new insights into PEC adipic acid synthesis via solar-driven processes and shows great implications for advanced technology that couples energy storage with value-added chemicals synthesis.

## Supplementary Material

nwag236_Supplemental_File

## Data Availability

All data supporting the findings of this study are available in the article and its Supplementary information.
