# Supplementary material for: Photoelectrosynthesis of adipic acid coupled with energy storage in an open-loop flow battery
Source: Natl Sci Rev. 2026 Apr 22;13(14):nwag236. doi: 10.1093/nsr/nwag236 (PMC13411275; doi:10.1093/nsr/nwag236)
Supplement: nwag236_Supplemental_File [file nwag236_supplemental_file.pdf]

Supplementary Information

Photoelectrosynthesis of adipic acid coupled with energy storage in an open-loop flow battery

Shanshan Zhang[1], Lan Luo[2]*, Yayue Dai[1], Jiangrong Yang[1], Wangsong Chen[1], Yucong Miao[1], Nenghui Pan[1], Zhenhua Li[1]*, Mingfei Shao[1]*

[1]State Key Laboratory of Chemical Resource Engineering, College of Chemistry, Beijing University of Chemical Technology, Beijing 100029, China

[2]Tianjin Key Laboratory of Brine Chemical Engineering and Resource Eco-utilization, College of Chemical Engineering and Materials Science, Tianjin University of Science and Technology, Tianjin 300457, China

* Corresponding Author: L.L. (Email: LL99544039@tust.edu.cn), Z.L. (Email: LZH0307@mail.buct.edu.cn) and M.S. (Email: shaomf@mail.buct.edu.cn).

Table of Contents

Catalyst preparation 1

Characterization 1

Photoelectrochemical measurements 3

Photoelectrochemical cyclohexanone oxidation 4

Computational details 5

Supplementary Figures 7

Supplementary Tables 46

Supplementary Notes 48

Supplementary References 54

Catalyst preparation

Preparation of TiO_2 photoanode. TiO_2 nanorod array was prepared via a facile hydrothermal method [1]. As a typical run, 0.175 mL of titanium butoxide was dropwise added into a H_2O/HCl mix solution with equal volume (6 mL) of H_2O and HCl (36.5–38 wt.%) under continuous stirring. The resulting solution and the FTO substrate were transferred and sealed in a Teflon-lined stainless-steel autoclave (25 mL) and heated to 150 °C for 20 h. The as-obtained TiO_2 nanorod array was removed from the autoclave, washed thoroughly with distilled water and dried in air, then followed by annealing at 350 °C in air for 2 h to improve the crystallinity of TiO_2 nanorods and enhance their contact to the FTO substrate.

Preparation of $NiFeCu(OH)_2/TiO_2$ photoanode. $NiFeCu(OH)_2/TiO_2$ photoanode catalyst was prepared by electrodeposition which the $Ni(NO_3)_2·6H_2O$ (7.48 mM), $FeSO_4·7H_2O$ (0.49 mM) and $Cu(NO_3)_2·3H_2O$ (2 mM) aqueous solution are used as the electrolyte. The electrodeposition adopts three-electrode configuration with pristine TiO_2 semiconductor as working electrode, saturated calomel electrode (SCE) and platinum electrode as reference electrode and opposite electrode respectively. The potentiostatic deposition was conducted at a potential of –1.0 V vs. SCE potential for 15 s. After the electrodeposition is complete, the electrodes are cleaned with distilled water for further electrochemical measurements.

Preparation of $NiFe(OH)_2/TiO_2$ photoanode. $NiFe(OH)_2/TiO_2$ photoanode was prepared via the same method as the above $NiFeCu(OH)_2/TiO_2$ photoanode except using $Ni(NO_3)_2·6H_2O$ (7.48 mM), $FeSO_4·7H_2O$ (2.52 mM) solution.

Characterization

Scanning electrode microscope (SEM) images were obtained by Zeiss Supra 55 field emission scanning electron microscope operated at 20.0 kV. Transmission electron microscopy (TEM) images were recorded with JEOL JEM-2010 high resolution (HR-)

TEM operated at 200 kV, combined with energy dispersive X-ray spectroscopy (EDX). Powder samples used in this work were scraped from the TiO_2, $NiFe(OH)_2/TiO_2$ and $NiFeCu(OH)_2/TiO_2$. X-ray powder diffraction (XRD) patterns were recorded using a Shimadzu XRD-6000 diffractometer equipped with a graphite-filtered Cu Kα radiation source (λ = 0.15418 nm). X-ray photoelectron spectra (XPS) were performed on a Thermo VG ESCALAB 250 X-ray photoelectron spectrometer at a pressure of about 2×10^{-9} Pa using Al Kα X-rays as excitation source. The metal loadings were confirmed by ICP optical emission spectrometry (ICP-OES) analysis (Perkin-Elmer 3300DV). UV-vis diffuse reflectance spectra were recorded on a UV-vis spectrophotometer (Shimadzu UV-3600, Japan) with an integrating sphere and with $BaSO_4$ as reference. The bandgap of samples was determined based on the Tauc plot. Photoluminescence (PL) spectra were obtained at ambient temperature using a HitachiF-7000 fluorescence spectrophotometer with an excitation wavelength of 300 nm. *In situ* Raman spectra were collected by HORIBA Jobin Yvon Raman microspectrometer (λ = 532 nm) using a 632 nm laser and the power was set at 2mW under different potentials monitored by a CHI 760E electrochemical workstation. Time of Flight Secondary Ion Mass. Spectrometry (TOF-SIMS) was conducted on TOF-SIMS 5-100 instrument (IONTOF GmbH) at energy for 30 keV and ion current for 0.75 pA. Electron paramagnetic resonance (EPR) spectra were collected on a JES-FA200 X ESR spectrometer (298 K, 9063,386 MHz) 5,5-Dimethyl-1-pyrroline N-oxide (DMPO) was used as the spin-trapping agent. 0.5 mg samples were dispersed in 2 mL cyclohexanone alkaline aqueous solution, then 50 μL DMPO was added into the solution, and after filled with argon irradiated with 300 W Xenon lamp (Microsolar 300; Beijing Perfectlight) with an AM 1.5G filter (100 mW cm^{-2}) for ESR measurement.

In situ DRFITs to investigate the adsorption of cyclohexanone on TiO_2, $NiFe(OH)_2/TiO_2$, $NiFeCu(OH)_2/TiO_2$ samples were recorded on a Nicolet iS50 spectrometer equipped with a cell fitted with BaF_2 windows and an MCT-A detector cooled with liquid nitrogen. The spectrum was collected at a resolution of 4 cm^{-1} with an accumulation of 64 scans in the range of 4000–1000 cm^{-1}. The samples powders

were scraped from the FTO and then filled into a self-supported wafer in each measurement. Then, the sample wafer was pretreated under a flow of Ar for 30 min at room temperature to remove the impurities absorbed on the surface. The background was collected at room temperature under a flow of Ar. CYC was bubbled into the sample by flowing Ar at room temperature for 30 min to obtain a stable spectrum, respectively. The acquisition time of each scan in the FT-IR spectra was 30 s. Then, the system was purged with a flow of Ar, and the desorption spectra were recorded toward the desorption time until there was no change in the band intensity.

Photoelectrochemical measurements

Photoelectrochemical measurements were conducted on an electrochemical workstation (CHI 760E, CH Instruments, Inc.) in a three-electrode system with an H-type cell, in which SCE electrode electrode as reference electrode, Pt foil as counter electrode, and photoanode was adopted as the working electrode (the working area is 1.0 cm^2). The simulated solar illumination was obtained from a 300 W Xenon lamp with an AM 1.5G filter (100 mW cm^{-2}). For the linear sweep voltammetry (LSV) test, photocurrent was recorded from 0.1 to 1.4 V vs. RHE at a scan rate of 10 mV s^{-1} in 0.5 M KOH. All potentials mentioned in this work were converted to potentials versus RHE (in volts) according to eq 1:

$$E_{RHE} = E_{SCE} + E_{SCE\ vs.\ NHE} + 0.059\ pH \quad (1)$$

where $E_{SCE\ vs.\ NHE}$ in equation 1 is 0.241 V at 20 °C.

PEC measurements with Na_2SO_3 as a hole scavenger were recorded in 0.5 M KOH with the addition of 0.5 mol L^{-1} Na_2SO_3. The efficiency of charge injection (η_{inj}) was calculated by eq 2:

$$\eta_{inj} = \frac{J_{H_2O}}{J_{Na_2SO_3}} \quad (2)$$

In which J_{H_2O} is the photocurrent density obtained in the PEC water oxidation experiment, while $J_{Na_2SO_3}$ is the photocurrent density obtained in the PEC measurements with Na_2SO_3 as a hole scavenger.

Photoelectrochemical cyclohexanone oxidation

Photoelectrochemical oxidative of cyclohexanone reaction was conducted in a sealed quartz cell at atmospheric pressure. Typically, the photoanode was immersed in 0.5 M KOH containing 50 mM cyclohexanone under continuous stirred. Then, the oxidative of cyclohexanone was performed at a potential of 1.0 V vs. RHE for 2 h under AM 1.5G, 100 mW cm^{-2} illumination at room temperature. The products of PEC CYC oxidation were analyzed by high performance liquid chromatography (HPLC; Angilent 1260) equipped with organic acid column (Coregel 87H3) and a UV detector (210 nm). The reactants (cyclohexanone) were quantified by gas chromatography flame ionization detector (GC, Agilent 7890B with an HP-5 column and an FID detector). Quantification was done with an external standard calibration curve. The error bars correspond to the standard deviation of three independent measurements.

Selectivity of product was calculated based on:

$$\text{Selectivity} = \frac{\text{Content of corresponding product}}{\text{Consumption of reactant}} \times 100\% \qquad (3)$$

Production rate of products was calculated according to:

$$\text{Production rate} = \frac{\text{Content of corresponding product}}{t \times A} \qquad (4)$$

where t is the reaction time (h), and A is the area of electrode (cm^2).

Faradaic efficiency was calculated by:

$$\text{Faradaic efficiency} = \frac{n_{\text{products}} \times Z_{\text{products}} \times F}{Q_{\text{total}}} \times 100\% \qquad (5)$$

where Z_{products} was the number of holes required to oxidize cyclohexanone molecule to produce adipic acid (Z = 6). n_{products} was the mole number of adipic acid. F is the Faraday constant (96485 C mol^{-1}), and Q_{total} is the total charge.

The incident solar-to-adipic acid conversion efficiency, alternatively termed the quantum efficiency (QE) of adipic acid was calculated as follows:

$$\text{QE} = \frac{J_{\text{ph}} \times \frac{hc}{e} \times \text{FE}_{\text{adipic acid}}}{P \times \lambda} \times 100\% \qquad (6)$$

where J_{ph} is the photocurrent density (mA cm^{-2}), h is Planck's constant (6.62×10^{-34}

J·s), c is the speed of light (3.0×10^8 m s^{-1}), e is the electron charge (1.602×10^{-19} C), $FE_{adipic\ acid}$ is the Faradaic efficiency of adipic acid, P is the monochromatic incident light intensity (mW cm^{-2}), and λ is the wavelength of monochromatic light (nm).

IPCE was measured under monochromatic irradiation under one sun illumination (AM 1.5 G, 100 mW cm^{-2}) equipped with a monochromator at 1.0 V vs. RHE:

$$\text{IPCE} = \frac{(J_{\text{light}} - J_{\text{dark}}) \times \frac{hc}{e}}{\text{P} \times \lambda} \times 100\% \quad (7)$$

where J_{light} is the photocurrent density under illumination, J_{dark} is the current density under dark condition.

Carbon efficiency was calculated as follows:

$$\eta_{\text{c}} = \frac{\text{n}_{\text{c, in product}} + \text{n}_{\text{c, remaning of reactant}}}{\text{n}_{\text{c, in feedstock}}} \times 100\% \quad (8)$$

where I_{out} is the output (discharging) current, V_{out} is the output voltage, S is the total incident solar irradiance, which is provided by the EKE-type light source at 100 mW cm^{-2}, and A is the illumination area of photoelectrode.

The solar-to-chemical (η_{STC}) was calculated based on Eq. 9:

$$\eta_{\text{STC}}\ (\%) = \frac{j_{\text{pc}} \times |\text{E}^0\text{-E}|}{\text{P}_{\text{in}}} \times 100\% \quad (9)$$

where j_{pc} is the photocurrent density at each applied bias (mA cm^{-2}), E is the applied bias potential (V), P_{in} is the incident solar power (AM 1.5G, 100 mA cm^{-2}).

Energy efficiency (EE) was calculated based on Eq. 10:

$$\eta_{\text{EE}}\ (\%) = \frac{\text{E}_{\text{discharge}}}{\text{E}_{\text{charge}}} \times 100\% \quad (10)$$

Computational details

The bulk models of $NiFe(OH)_2$ and $NiFeCu(OH)_2$ were constructed with the P3m1 space group. The $NiFe(OH)_2$ model contains 7 Fe, 9 Ni, 32 H and 32 O atoms; the $NiFeCu(OH)_2$ model contains 10 Fe ,3 Ni, 3 Cu, 32 H and 32 O atoms. For both $NiFe(OH)_2$ and $NiFeCu(OH)_2$, a (4 × 1) supercell slab of the (100) plane, containing four layers, was constructed with a 20 Å vacuum space in the z-direction in order to avoid the spurious image-image interactions.

All spin-polarized density functional theory (DFT) calculations were performed

using Vienna Ab-initio Simulation Package (VASP) [2-4]. The exchange-correlation functional was approximated using the generalized gradient approximation with the Perdew-Burke-Ernzerhof (PBE) functional [5]. Projected-augmented wave (PAW) pseudopotentials were employed to describe the core-valence interactions [6]. The Hubbard U correction was included to partly account for the strong correlation in the 3*d* orbitals of Fe and Ni, with the U values of 4.3 and 5.5 eV, respectively [7]. The DFT-D3 method proposed by Grimme et al. was used to account for the van der Waals (vdW) corrections [8]. The plane-wave cutoff energy was set to 450 eV. Brillouin-zone sampling was executed with a Γ-centered 3 × 1 × 1 and 5 × 1 × 1 Monkhorst-Pack *k*-point grids for geometry optimization and electronic structure calculations, respectively. Gas-phase molecules were calculated using only the Γ point. All structures were fully optimized with convergence thresholds of 10^{-6} eV for electronic relaxation and 0.05 eV/Å for Hellmann-Feynman force on each atom.

The adsorption energies (E_{ads}) of CYC were computed as following equation:

$$E_{ads} = E_{CYC*} - E_{CYC} - E_{*} \quad (11)$$

where E_{CYC*}, E_{CYC}, and E_{*} represent the total energies of CYC adsorbed on the $NiFe(OH)_2$ and $NiFeCu(OH)_2$ surfaces, the CYC, and the clean surfaces, respectively.

Supplementary Figures

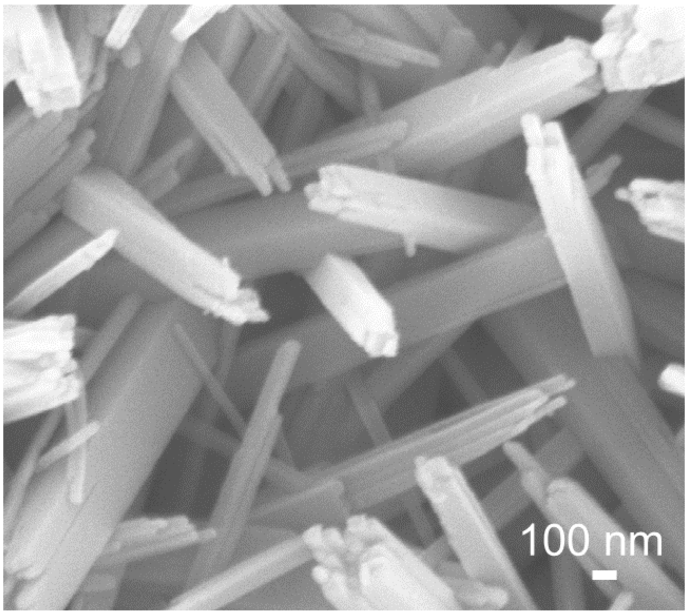

Fig. S1 SEM image. SEM image of the as-prepared TiO_2 photoanode.

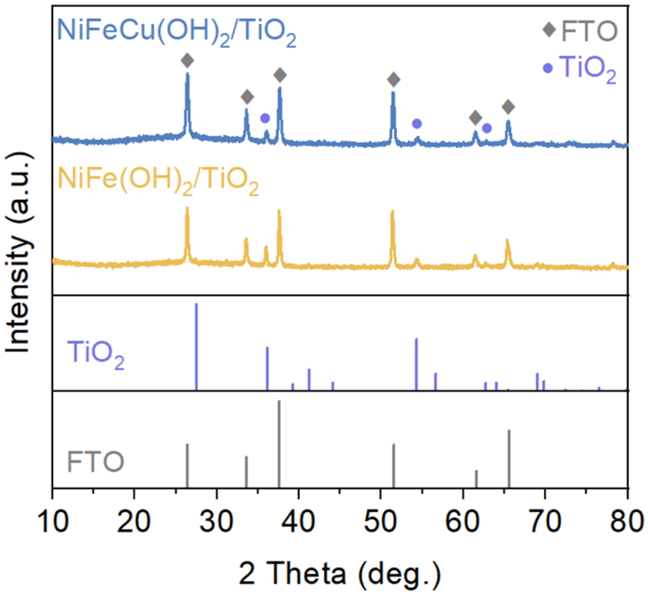

Fig. S2 XRD characterizations. XRD patterns of $NiFe(OH)_2/TiO_2$ and $NiFeCu(OH)_2/TiO_2$ samples on FTO substrate. a.u.: arbitrary units.

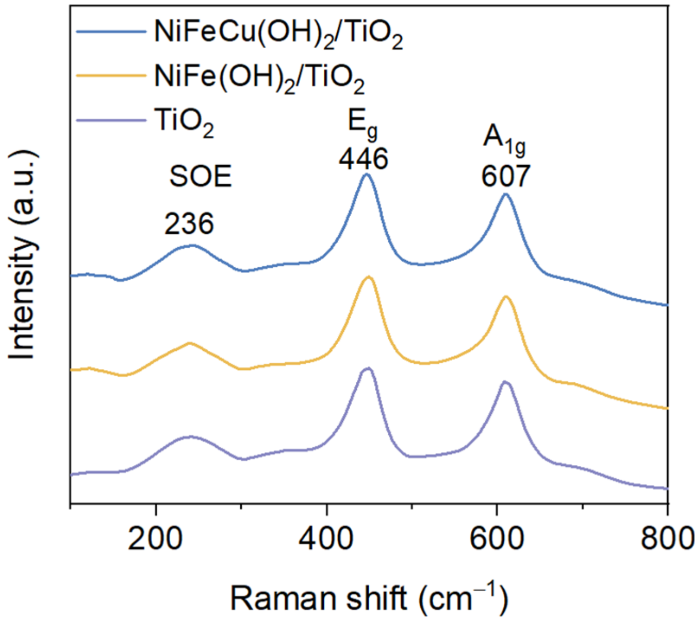

Fig. S3 Raman spectra. Raman spectra of TiO_2, $NiFe(OH)_2/TiO_2$, and $NiFeCu(OH)_2/TiO_2$ samples. a.u.: arbitrary units.

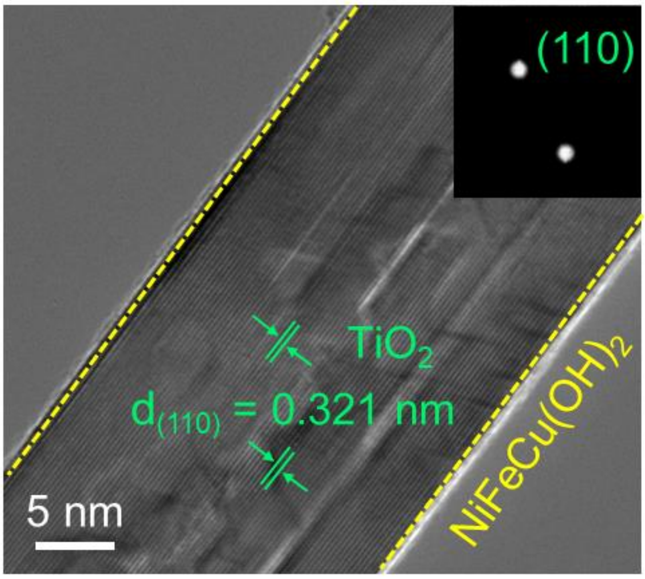

Fig. S4 HRTEM images. HRTEM images of $NiFeCu(OH)_2/TiO_2$ photoanode. Scale bar: 5 nm.

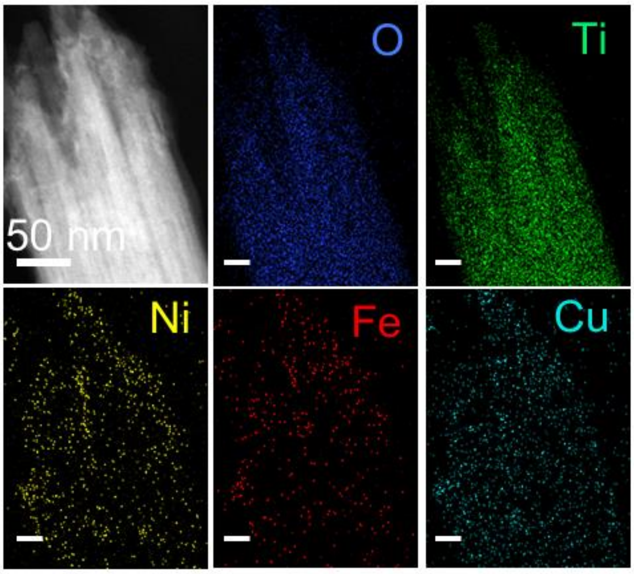

Fig. S5 STEM-EDS mapping. STEM-EDS mapping results of NiFeCu(OH)$_2$/TiO$_2$ photoanode for O, Ti, Ni, Fe, and Cu elements. Scale bars: 50 nm.

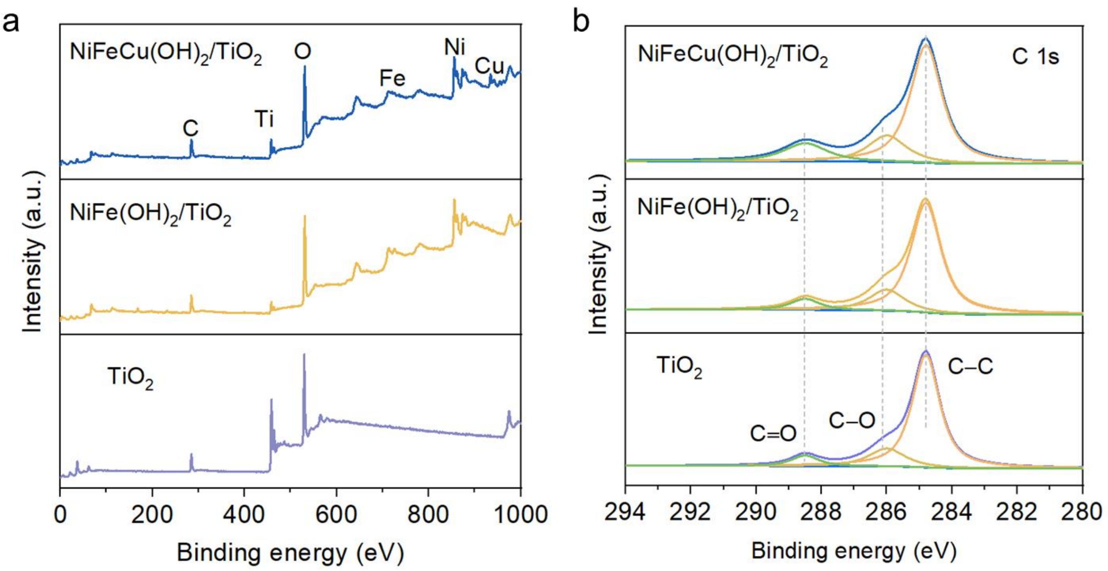

Fig. S6 Characterizations of electronic structure. (a) XPS survey spectra and (b) C 1s XPS spectra of TiO_2, $NiFe(OH)_2/TiO_2$, and $NiFeCu(OH)_2/TiO_2$ photoanodes. a.u.: arbitrary units.

The C 1s XPS spectra of TiO_2, $NiFe(OH)_2/TiO_2$, and $NiFeCu(OH)_2/TiO_2$ samples exhibit three characteristic peaks at 284.8, 286.0, and 288.5 eV, which are attributed to C−C, C−O, and C=O bonds, respectively.

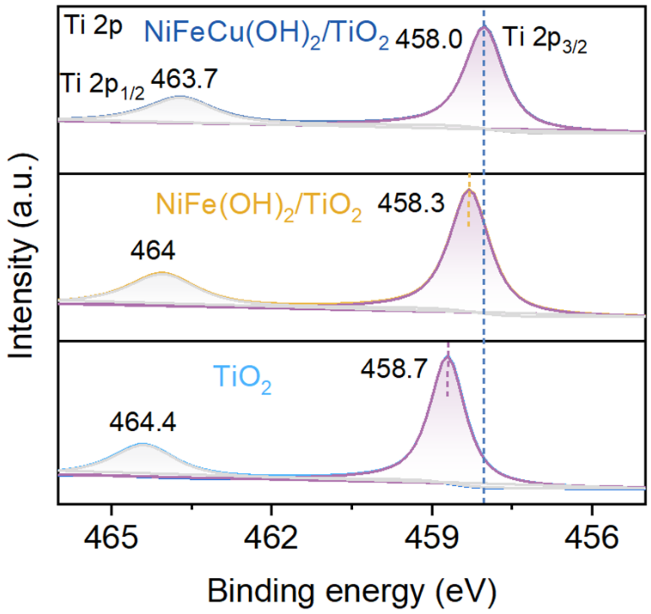

Fig. S7 Characterizations of electronic structure. High-resolution Ti 2p XPS spectra of TiO_2, $NiFe(OH)_2/TiO_2$, and $NiFeCu(OH)_2/TiO_2$ samples. a.u.: arbitrary units.

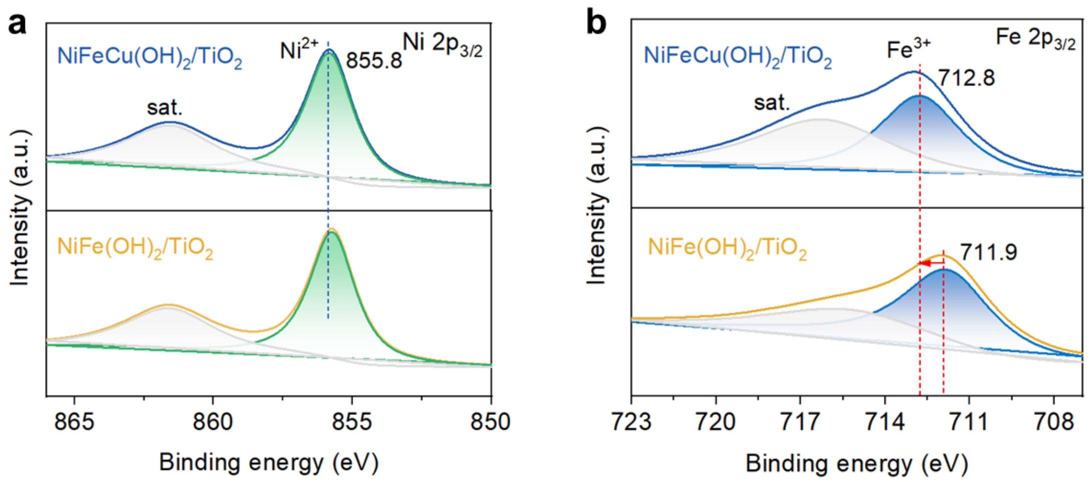

Fig. S8 Characterizations of electronic structure. High-resolution (a) Ni $2p_{3/2}$ XPS and (b) Fe $2p_{3/2}$ data for $NiFeCu(OH)_2/TiO_2$ photoanode (top) and $NiFe(OH)_2/TiO_2$ photoanode (bottom).

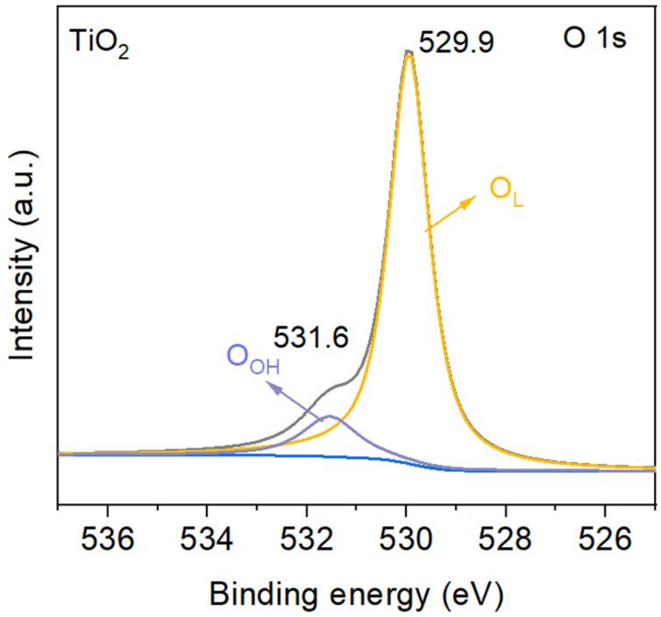

Fig. S9 Characterizations of electronic structure. High-resolution O 1s XPS spectra of TiO_2 sample. a.u.: arbitrary units.

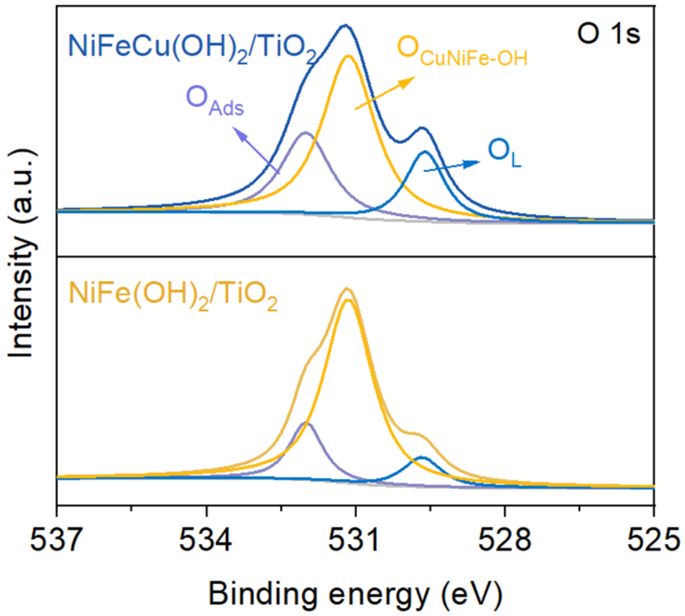

Fig. S10 Characterizations of electronic structure. High-resolution O 1s XPS spectra of $NiFe(OH)_2/TiO_2$ and $NiFeCu(OH)_2/TiO_2$ samples. a.u.: arbitrary units.

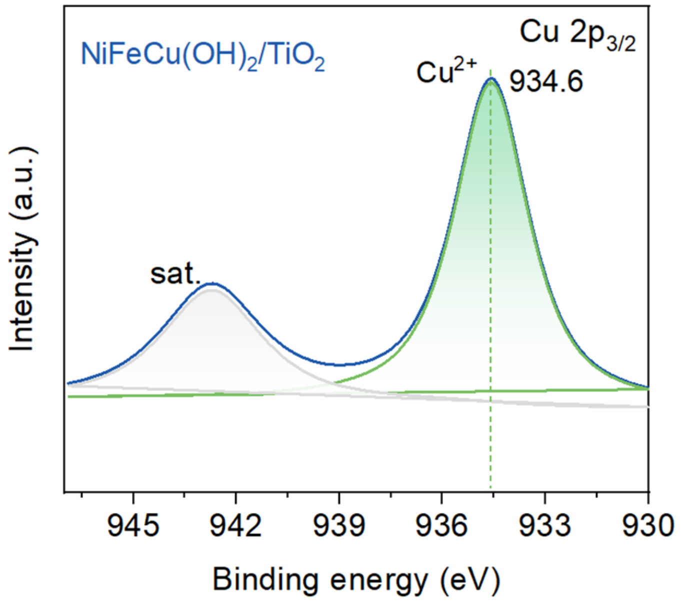

Fig. S11 Characterizations of electronic structure. High-resolution Cu $2p_{3/2}$ XPS spectra of $NiFeCu(OH)_2/TiO_2$ sample. a.u.: arbitrary units.

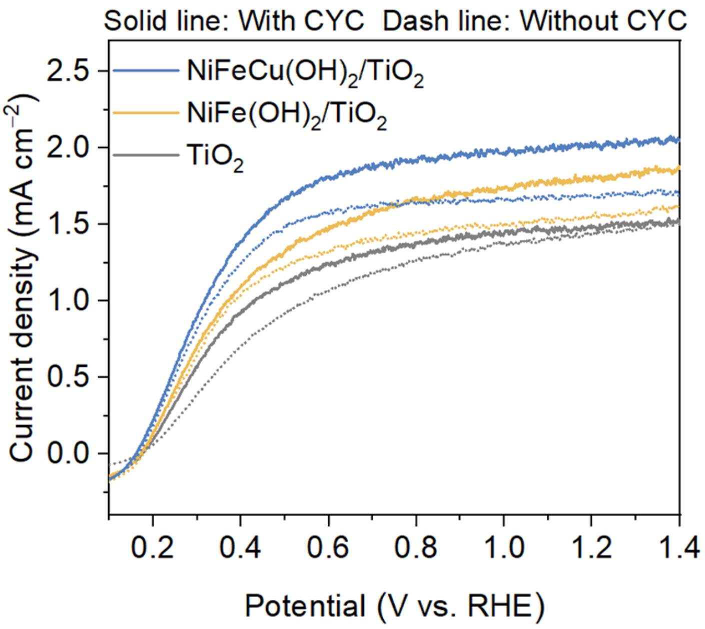

Fig. S12 Photocurrent measurements. LSV curves of different samples at a scan rate of 10 mV s^{-1} in 0.5 M KOH with or without 50 mM CYC under AM 1.5 G (100 mW cm^{-2}) illumination.

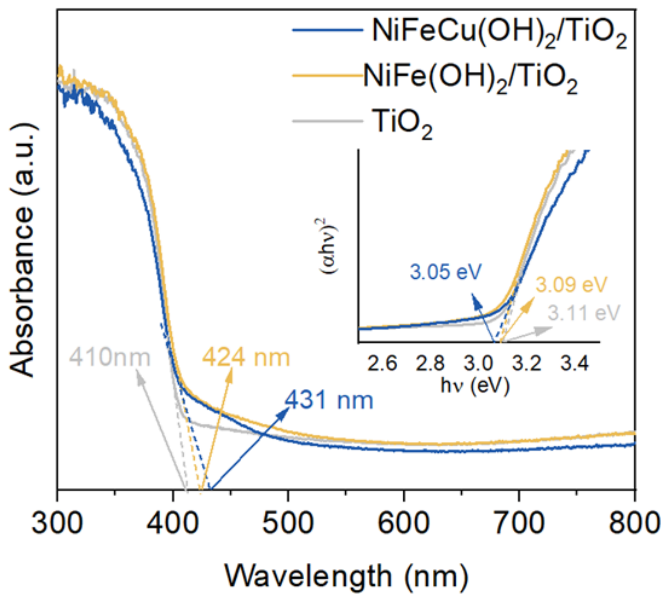

Fig. S13 Characterizations of light absorption. UV-vis diffuse reflectance spectra of NiFeCu$(OH)_2/TiO_2$, NiFe$(OH)_2/TiO_2$, and TiO_2 photoanodes. Inset shows the corresponding $(\alpha h\nu)^{1/2}$ versus photon energy plot.

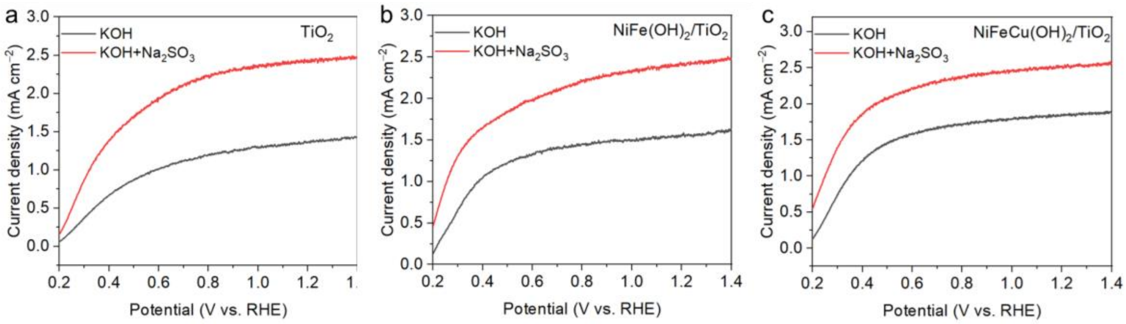

Fig. S14 Measurements of injection efficiency. LSV curves of (a) TiO_2, (b) NiFe$(OH)_2/TiO_2$ and (c) NiFeCu$(OH)_2/TiO_2$ photoanodes in 0.5 M KOH with and without 0.5 M Na_2SO_3.

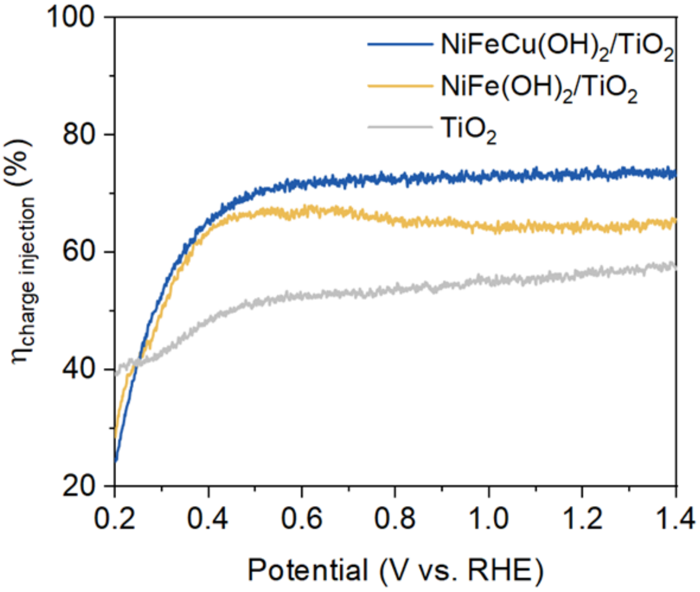

Fig. S15 Measurements of injection efficiency. Charge injection efficiency of $NiFeCu(OH)_2/TiO_2$, $NiFe(OH)_2/TiO_2$, and TiO_2 photoanodes.

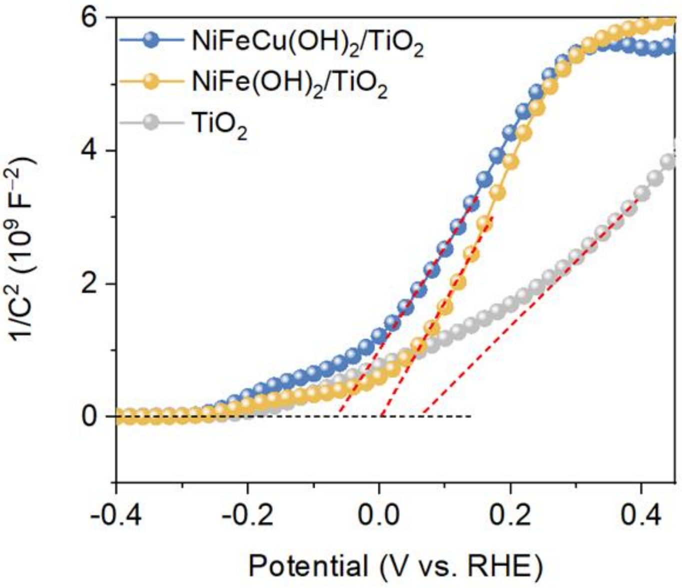

Fig. S16 Analysis of charge transport properties. Mott-Schottky plots of $NiFeCu(OH)_2/TiO_2$, $NiFe(OH)_2/TiO_2$, and TiO_2 photoanodes.

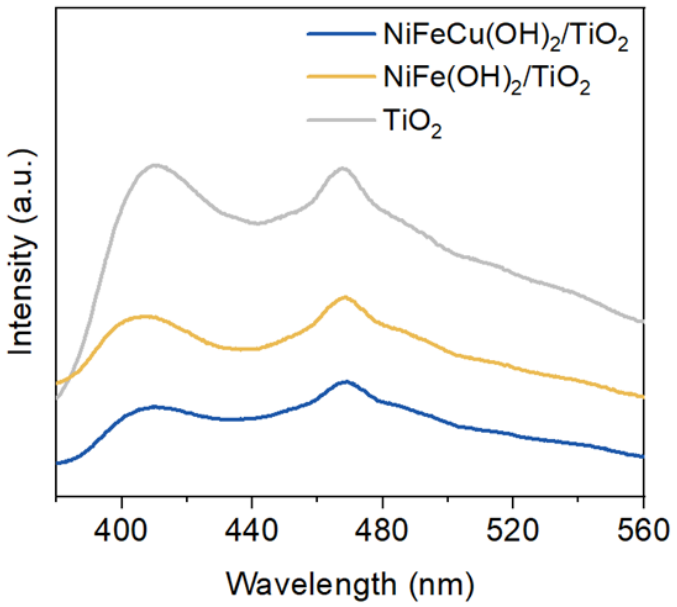

Fig. S17 Analysis of charge kinetics. PL spectra of $NiFeCu(OH)_2/TiO_2$, $NiFe(OH)_2/TiO_2$, and TiO_2 photoanodes.

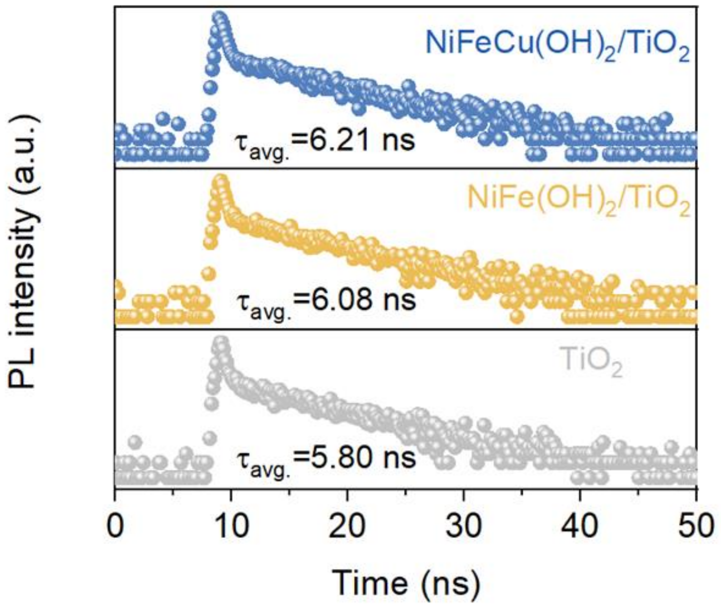

Fig. S18 Characterizations of carrier lifetimes. TRPL spectra (inset numbers are the average charge lifetime of each sample) of $NiFeCu(OH)_2/TiO_2$, $NiFe(OH)_2/TiO_2$, and TiO_2 photoanodes.

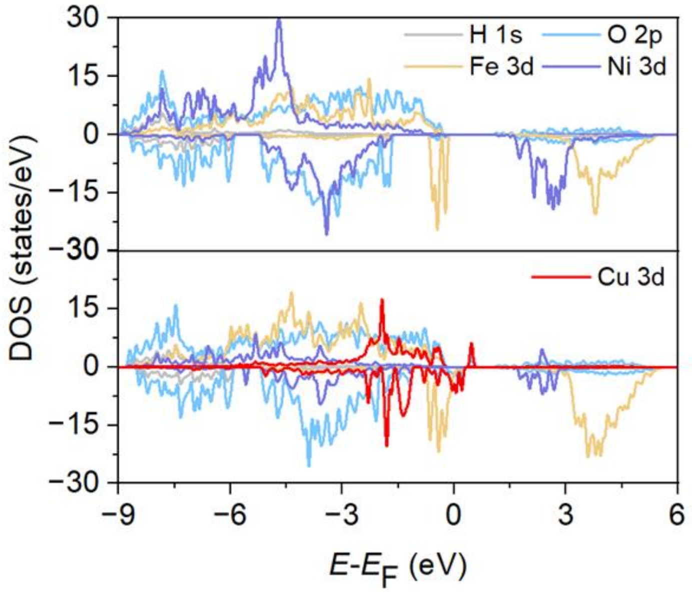

Fig. S19 Calculation. DOS analysis of $NiFeCu(OH)_2$ and NiFe $(OH)_2$. The 0 eV represent the Fermi energy (E_F) level.

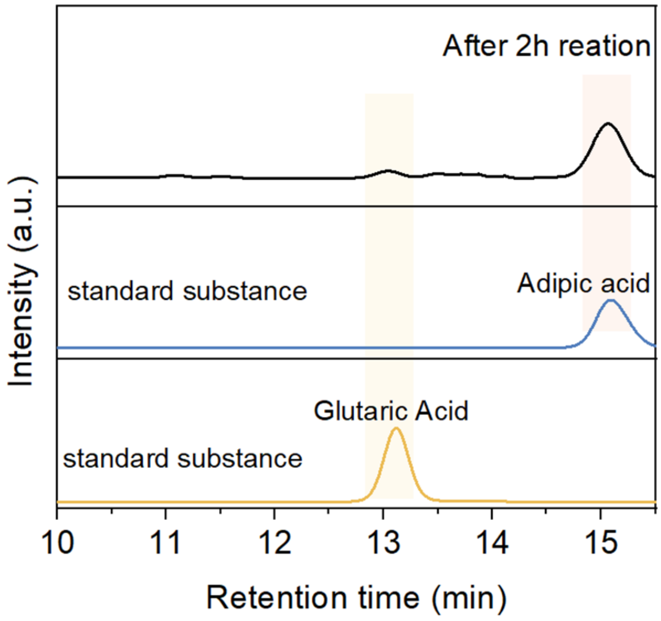

Fig. S20 Products quantification. High performance liquid chromatography (HPLC) spectra of the PEC CYC oxidation products over $NiFeCu(OH)_2/TiO_2$ photoanode under AM 1.5 G (100 mW cm^{-2}) illumination for 2 h.

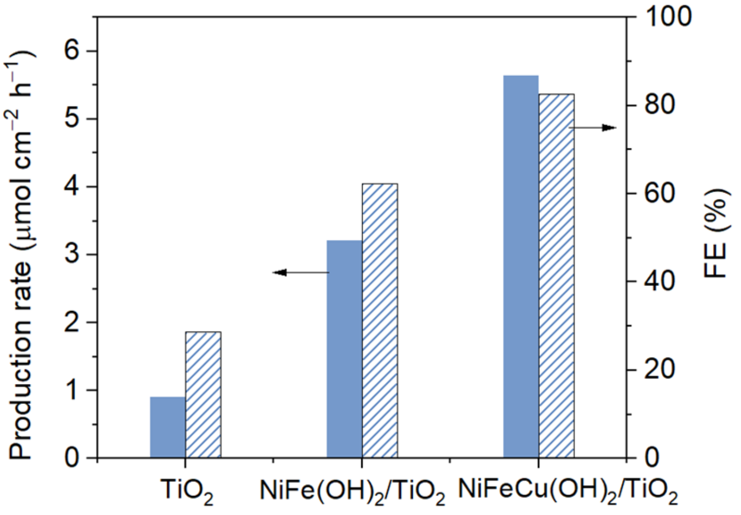

Fig. S21 The performance of PEC CYC oxidation over different photoanodes. Production rate and FE of adipic acid at 1.0 V vs. RHE over $NiFeCu(OH)_2/TiO_2$, $NiFe(OH)_2/TiO_2$, and pristine TiO_2 photoanodes under illumination.

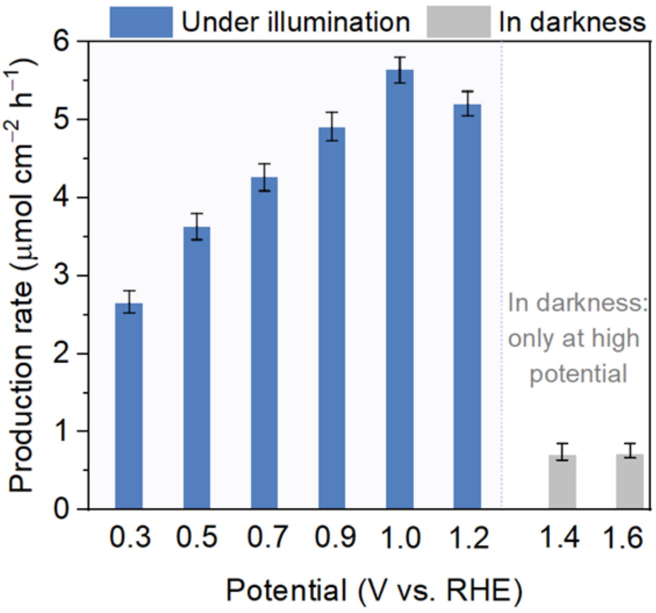

Fig. S22 The performance of PEC CYC oxidation over different potentials. Production rate of adipic acid at different potentials (0.3-1.6 V vs. RHE) over $NiFeCu(OH)_2/TiO_2$ photoanode under illumination or dark conditions.

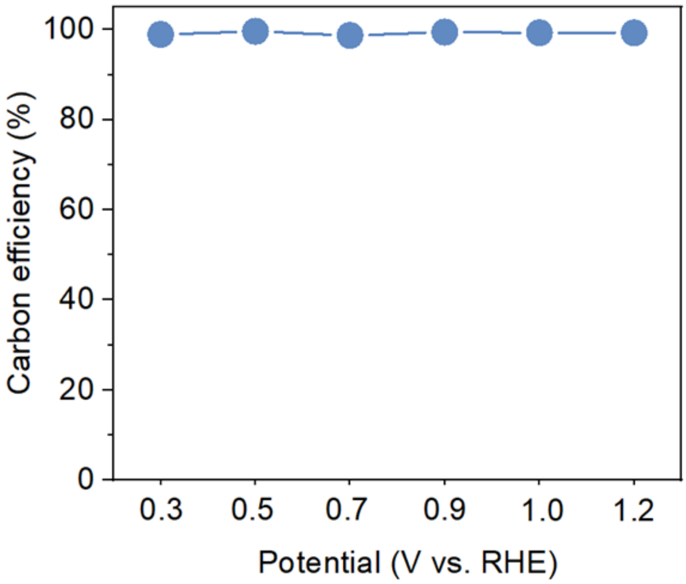

Fig. S23 Carbon efficiency. Carbon efficiency over $NiFeCu(OH)_2/TiO_2$ photoanode under illumination at different potentials (0.3-1.2 V vs. RHE).

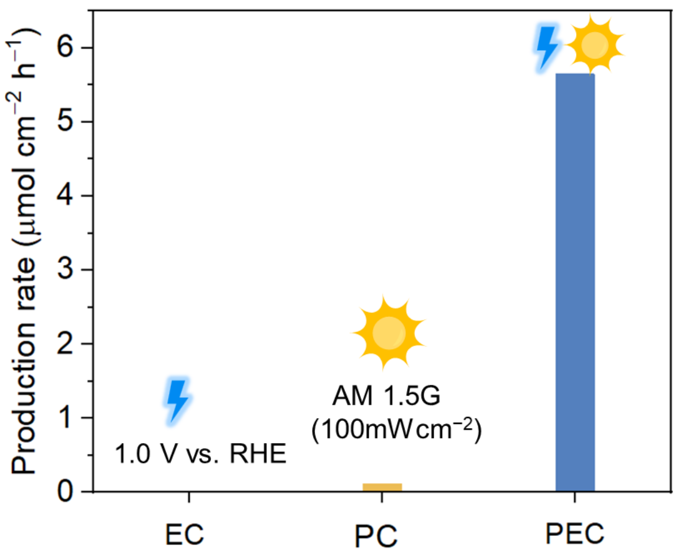

Fig. S24 The performance of PEC CYC oxidation over different conditions. Production rate of adipic acid over NiFeCu(OH)$_2$/TiO$_2$ photoanode in 0.5 M KOH electrolyte with 50 mM CYC at 1.0 V vs. RHE in dark (Electrocatalysis, EC) or only under AM 1.5G (100 mW cm^{-2}) illumination (Photocatalysis, PC), respectively.

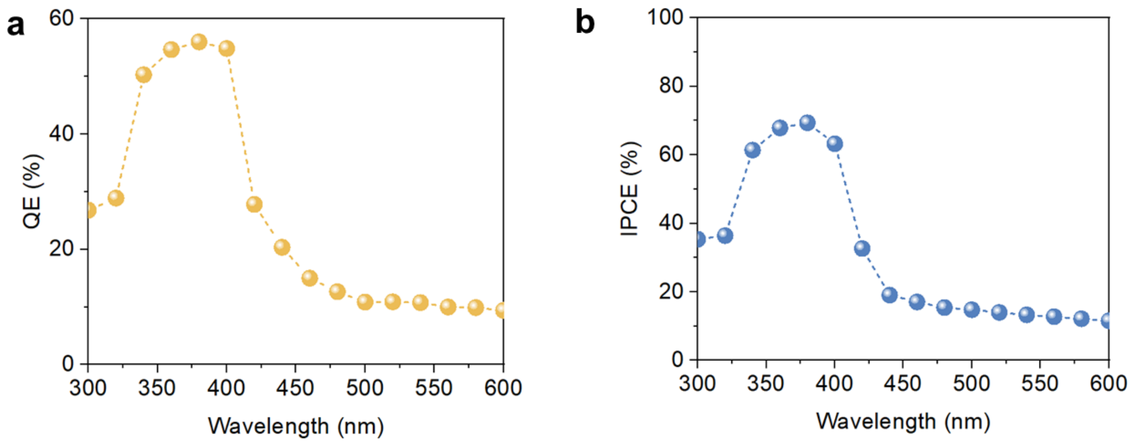

Fig. S25 QE and IPCE measurement. (a) Solar-to-adipic acid quantum efficiency (QE) and (b) photon-to-current conversion efficiencies (IPCE) spectra of NiFeCu$(OH)_2/TiO_2$ photoanodes acquired at 1.0 V vs. RHE in 0.5 M KOH with 50 mM CYC.

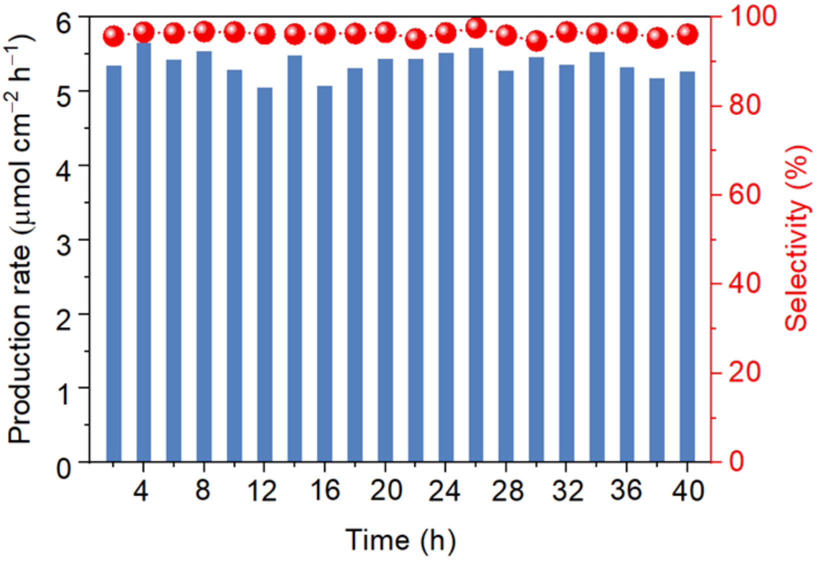

Fig. S26 The stability. The performance of long-term stability over NiFeCu$(OH)_2/TiO_2$ photoanode. Catalytic durability test of NiFeCu$(OH)_2/TiO_2$ in PEC CYC oxidation at 1.0 V vs. RHE for 40h (with 2 h for per batch).

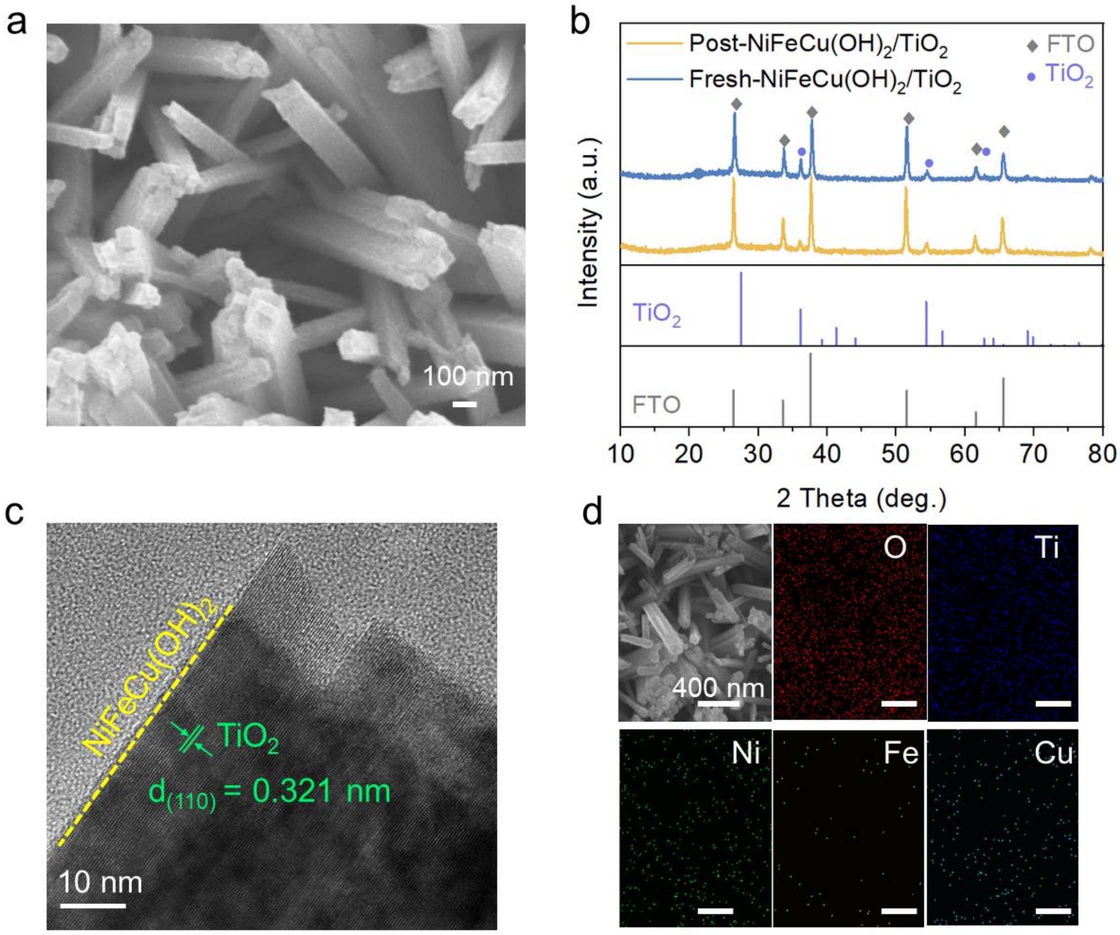

Fig. S27 The characterizations after batches tests. (a) SEM image, (b) XRD patterns, (c) HRTEM image, and (d) EDS mapping of used NiFeCu(OH)$_2$/TiO$_2$ photoanode after recycle stability test.

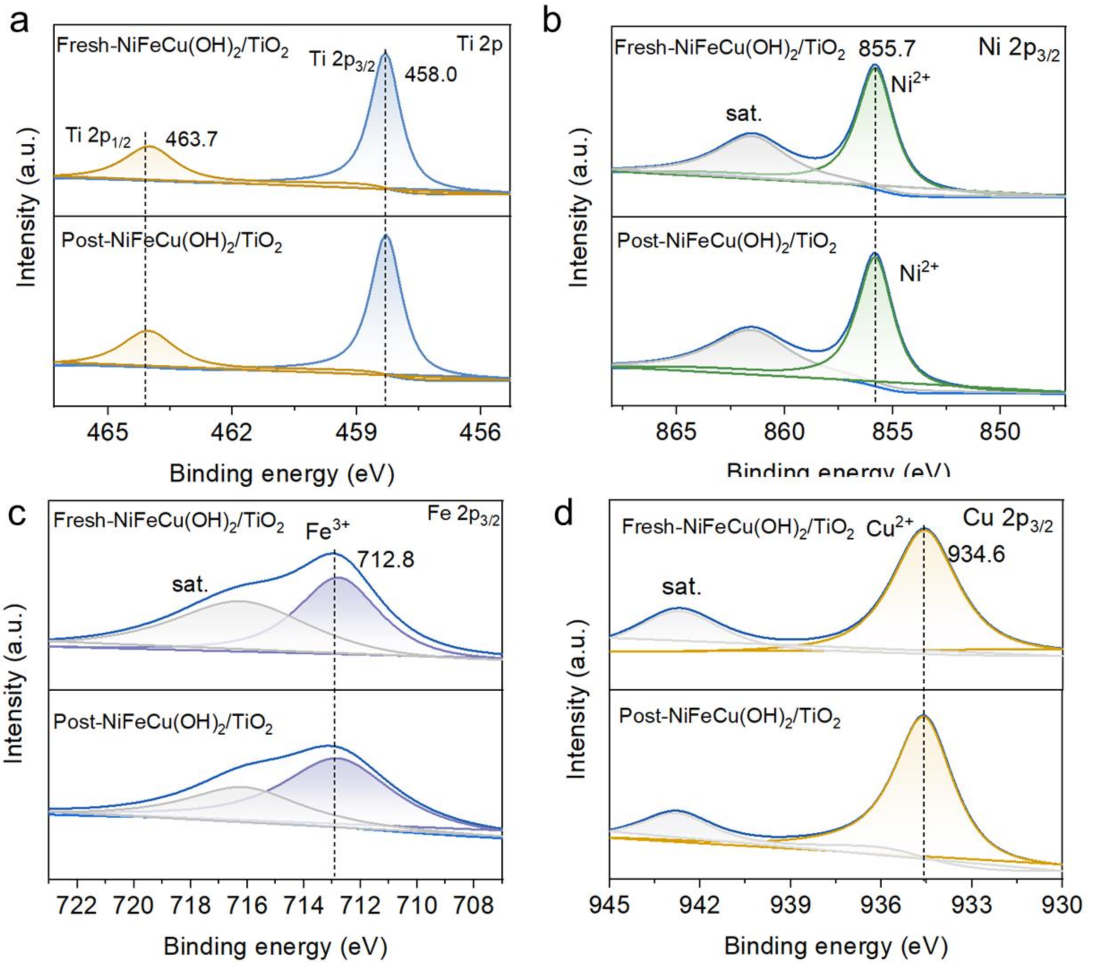

Fig. S28 The XPS after batches tests. (a) Ti 2p (b) Ni $2p_{3/2}$ (c) Fe $2p_{3/2}$ and (d) Cu $2p_{3/2}$ of fresh-NiFeCu$(OH)_2$/TiO_2 and post-NiFeCu$(OH)_2$/TiO_2 after recycle stability test.

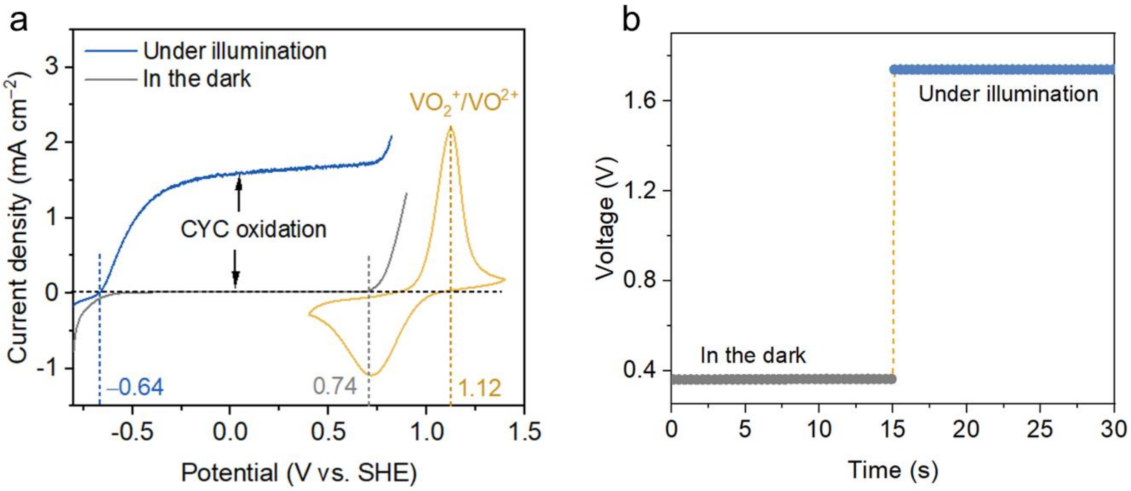

Fig. S29 The performance of PEC OLFB. (a) The LSV curves of CYC in 0.5 M KOH under AM 1.5G (100 mW cm^{-2}) illumination or dark conditions, and CV curve of VO_2^+/VO^{2+} redox couple. Dash line indicates the formal potential of CYC and the redox couple. (b) The OCV of the designed OLFB under both dark and illuminated conditions.

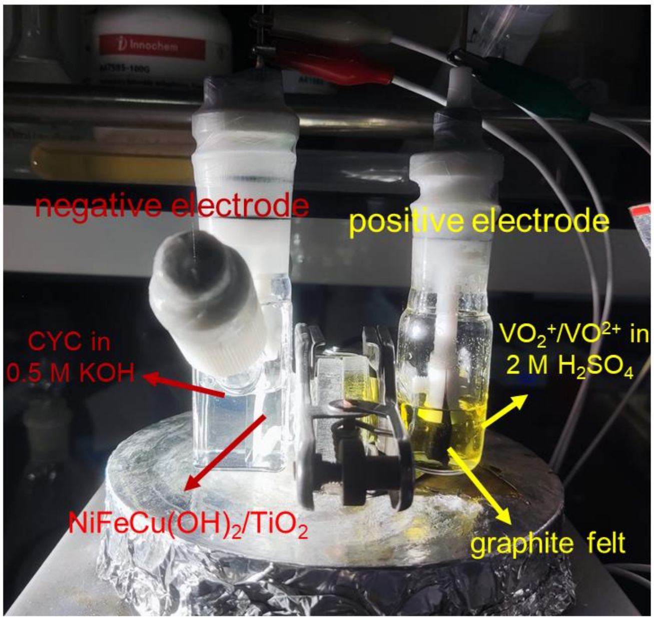

Fig. S30 The photo image of H-type quartz cell for PEC open-loop flow battery coupled with adipic acid synthesis with a two-electrode configuration. The $NiFeCu(OH)_2/TiO_2$ photoanode was used as the photoanode, and graphite felt as the positive electrode.

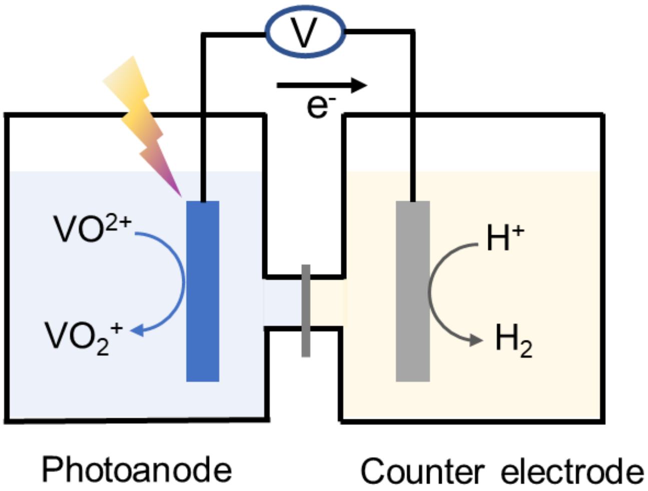

Fig. S31 Schematic illustration of charging. Schematic illustration of PEC open-loop flow battery for solar charging.

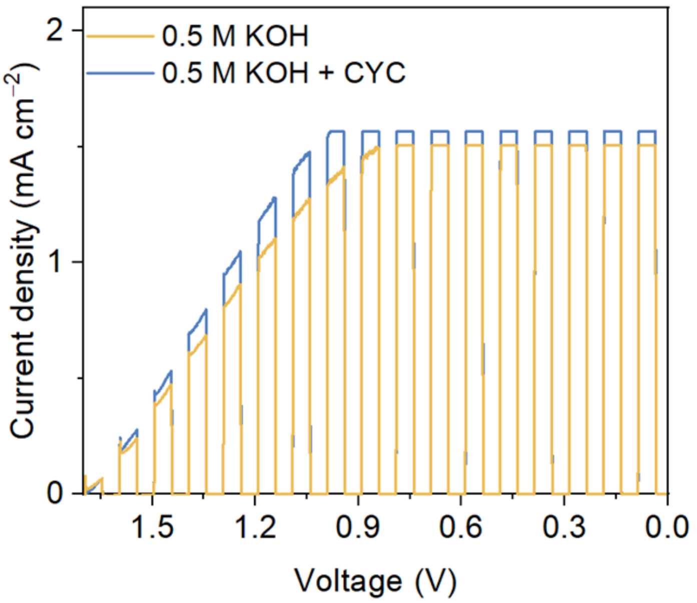

Fig. S32 Transient photocurrent measurement. Chopped polarization curves of the $NiFeCu(OH)_2/TiO_2$ photoelectrode and VO_2^+/VO^{2+} redox couple matched system.

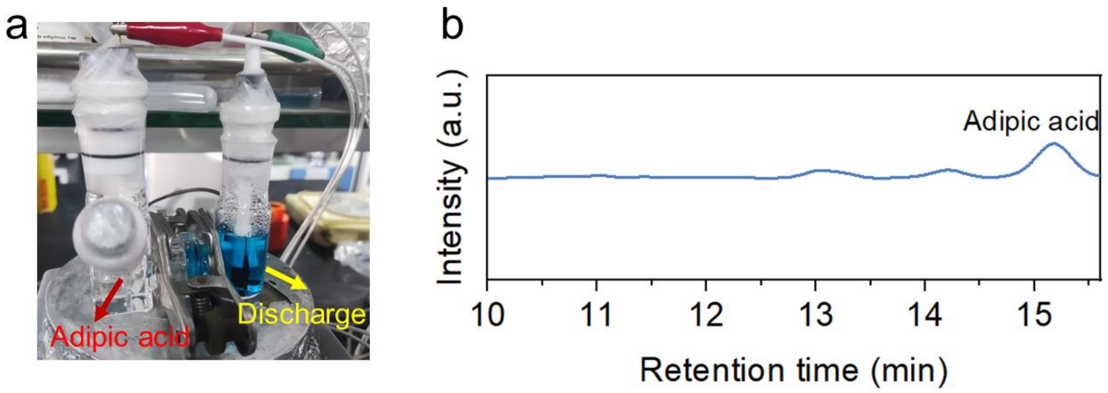

Fig. S33 The discharge process and adipic acid production. (a) The photo image of H-type quartz cell for PEC open-loop flow battery coupled with adipic acid synthesis after discharge. (b) HPLC spectra of products generated at the negative electrode after discharge in the PEC open-loop flow battery with 50 mM CYC in 0.5 M KOH as the negative electrolyte.

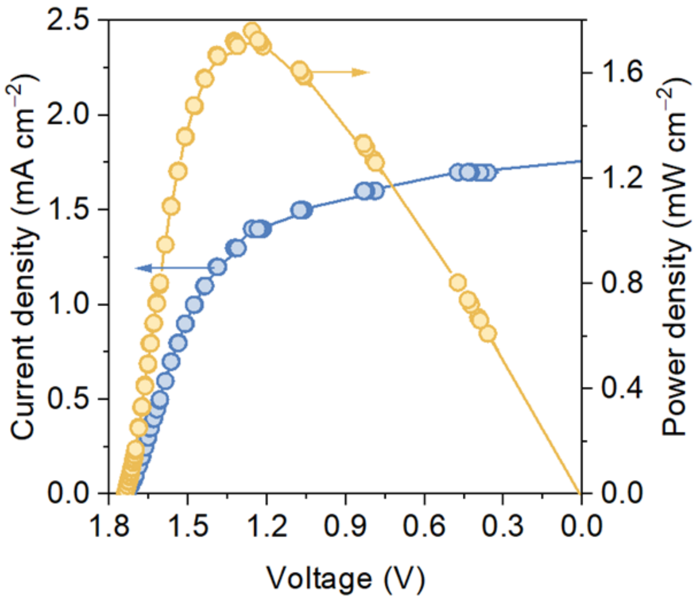

Fig. S34 Discharge performance. Polarization and power density curves of the PEC OLFB with/without CYC in 0.5 M KOH as the negative electrolyte in flow cell.

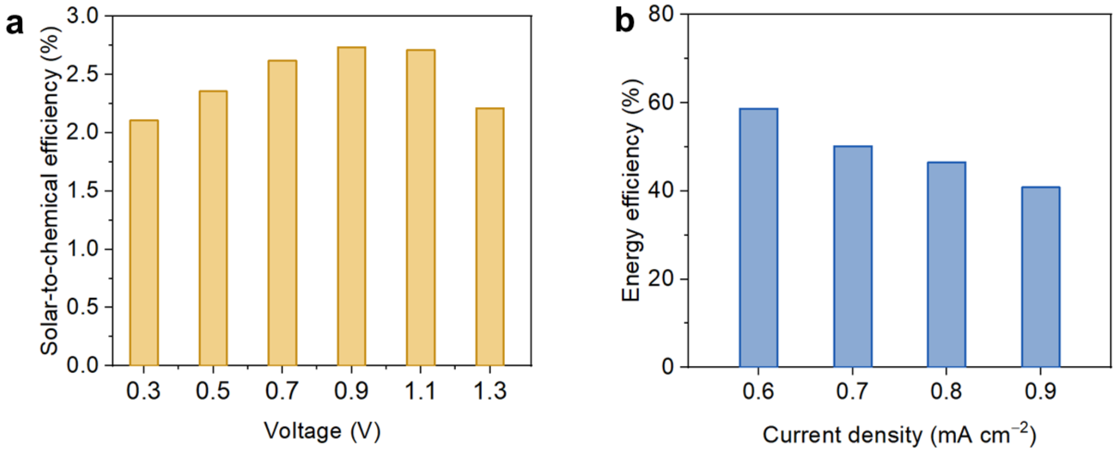

Fig. S35 PEC OLFB performance. (a) Solar-to-chemical conversion efficiency of PEC OLFB at different voltage (0.3-1.3 V vs. RHE). (b) PEC OLFB energy efficiency at different current densities.

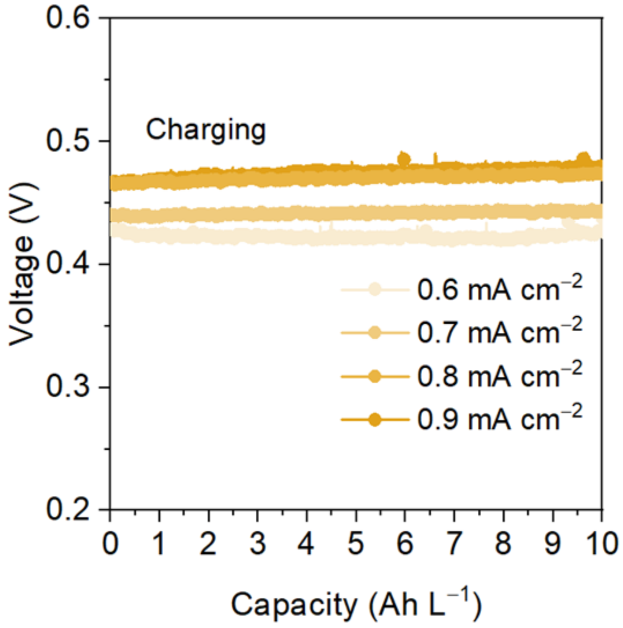

Fig. S36 Charge performance. Charge profiles performance of the PEC OLFB at different current densities.

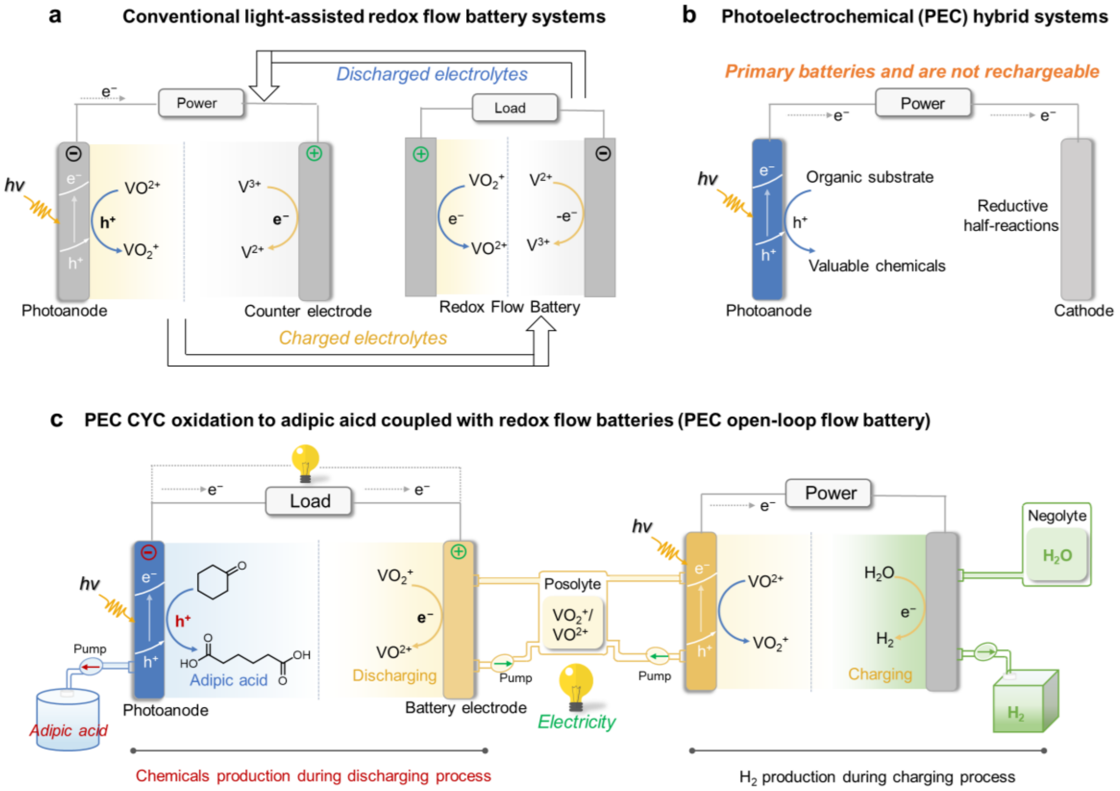

Fig. S37 Schematic diagram of the redox flow batteries. (a) Traditional light-assisted vanadium redox flow batteries. (b) PEC hybrid systems. (c) PEC open-loop flow battery coupled with hydrogen production and CYC oxidation (PEC OLFB; this work).

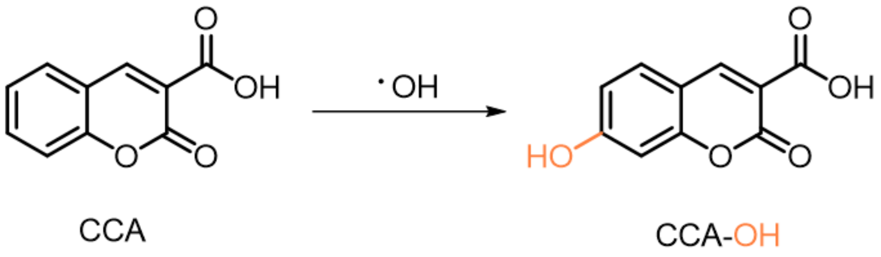

Fig. S38 Schematic diagram of CCA-OH. Schematic diagram illustrating the principle of CCA as probe molecule for capturing hydroxyl radicals.

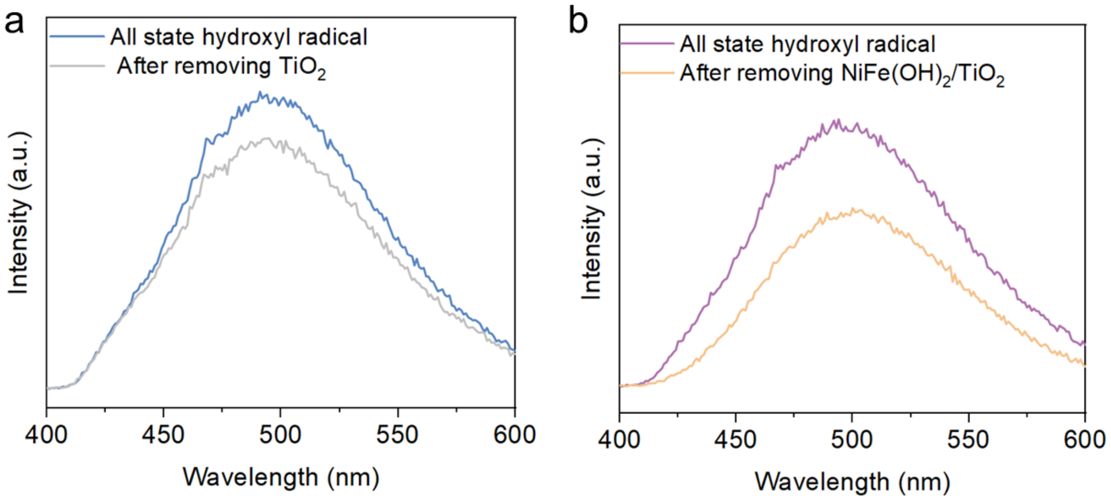

Fig. S39 Fluorescence spectra of (a) TiO_2, and (b) $NiFe(OH)_2/TiO_2$ for the detection of adsorbed OH* using CCA indicators.

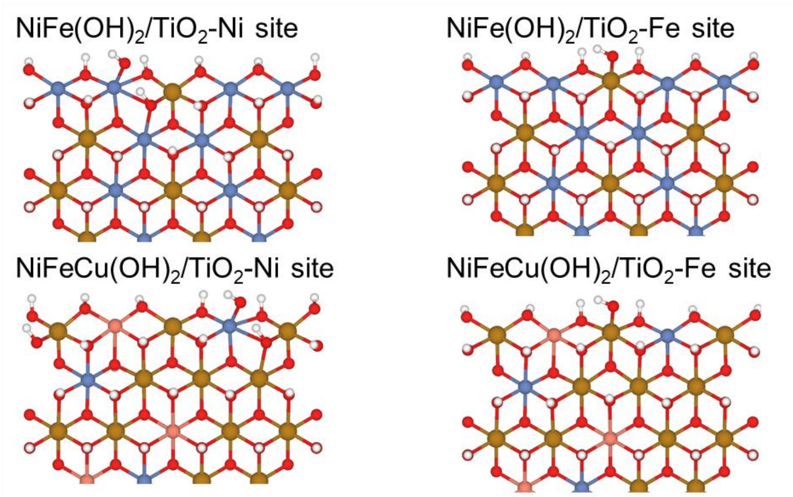

Fig. S40 The optimized OH* formation geometries. The color of each element is blue for Ni, brown for Fe, pink for Cu, red for O, and white for H, respectively.

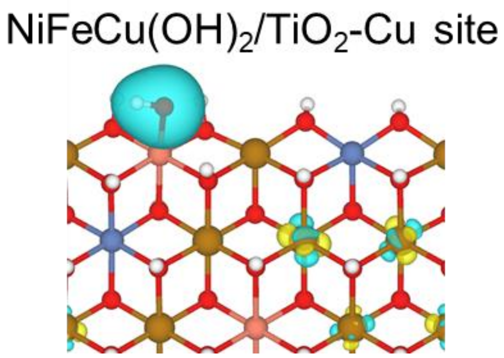

Fig. S41 Calculation. Charge density difference for OH* adsorption on Cu site for $NiFeCu(OH)_2/TiO_2$.

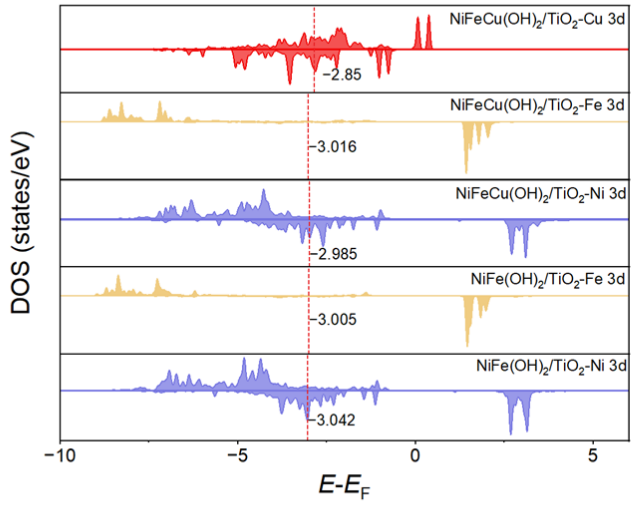

Fig. S42 Calculation. DOS analysis of $NiFeCu(OH)_2$ and NiFe $(OH)_2$. The 0 eV represent the Fermi energy (E_F) level.

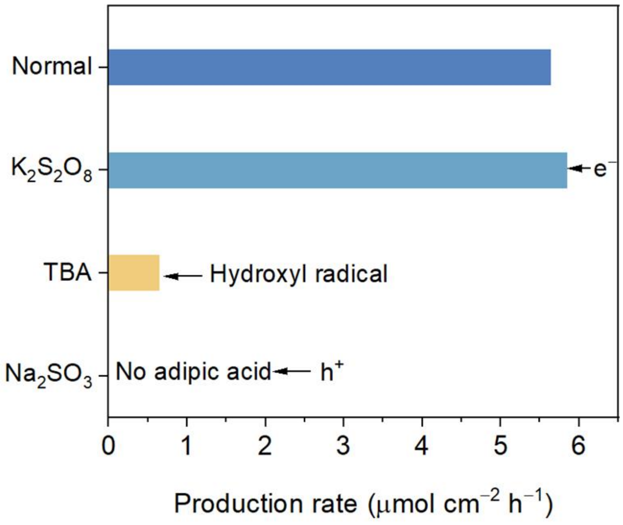

Fig. S43 Quenching experiments of PEC CYC oxidation. Na_2SO_3 as the hole scavenger, TBA as the hydroxyl radical scavenger, $K_2S_2O_8$ as the electron scavenger, Normal: no scavenger.

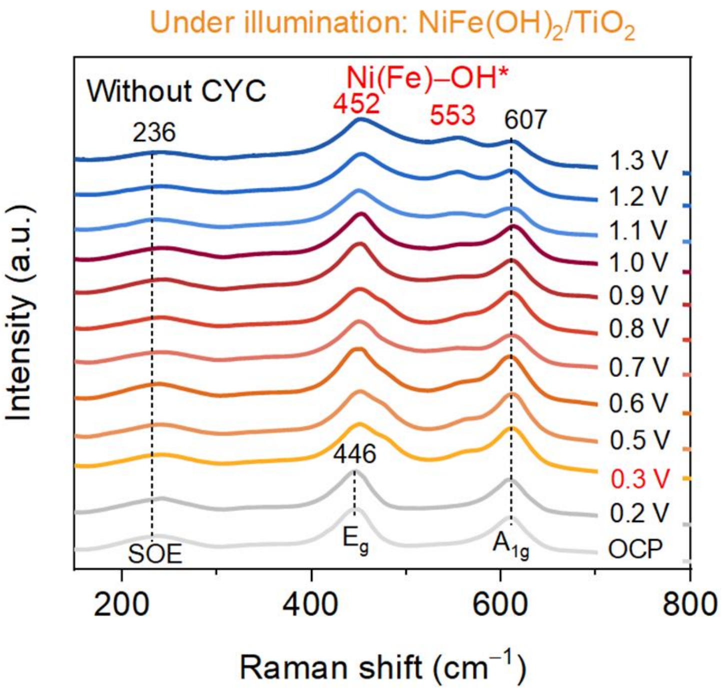

Fig. S44 In-situ Raman spectra. *In-situ* Raman spectra of $NiFe(OH)_2/TiO_2$ photoanode in 0.5 M KOH under illumination.

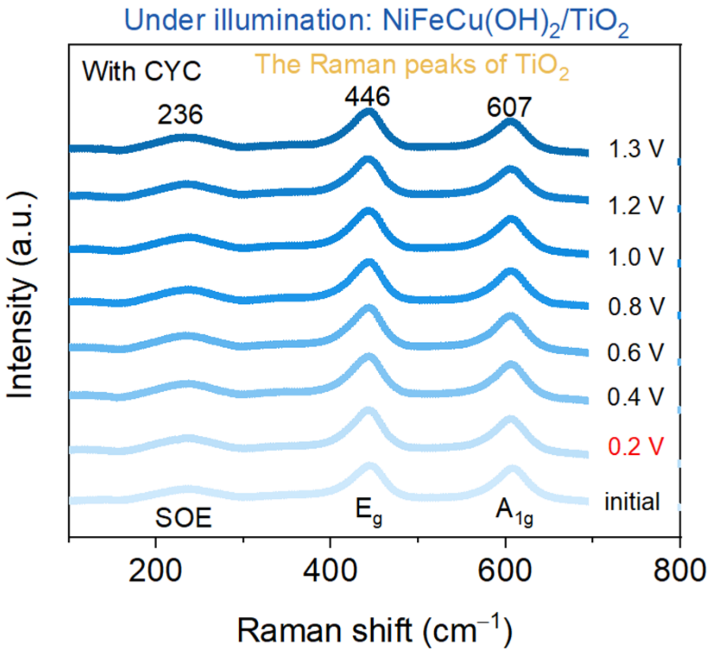

Fig. S45 *In-situ* Raman spectra. *In-situ* Raman spectra of $NiFeCu(OH)_2/TiO_2$ photoanode in 0.5 M KOH with 50 mM CYC under illumination.

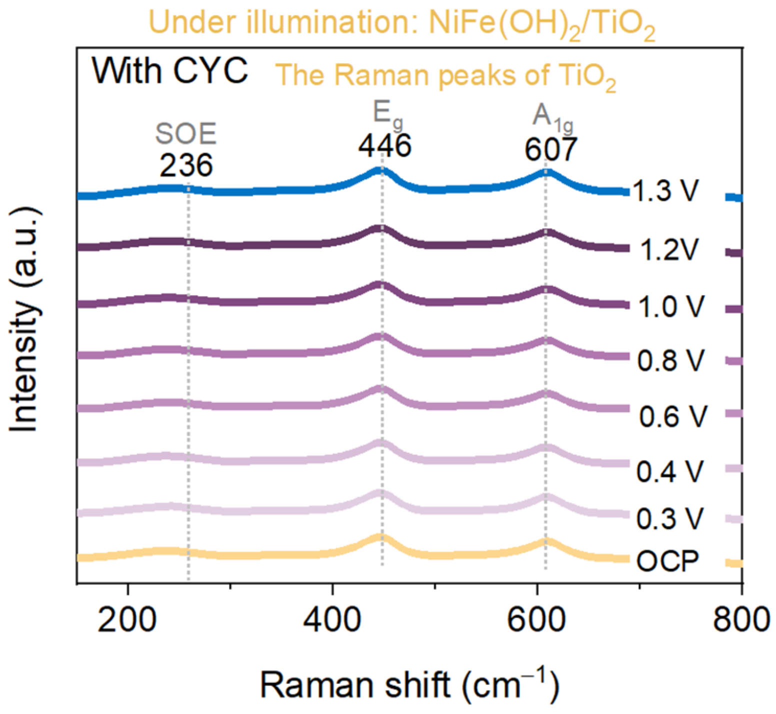

Fig. S46 *In-situ* Raman spectra. *In-situ* Raman spectra of $NiFe(OH)_2/TiO_2$ photoanode in 0.5 M KOH with 50 mM CYC under illumination.

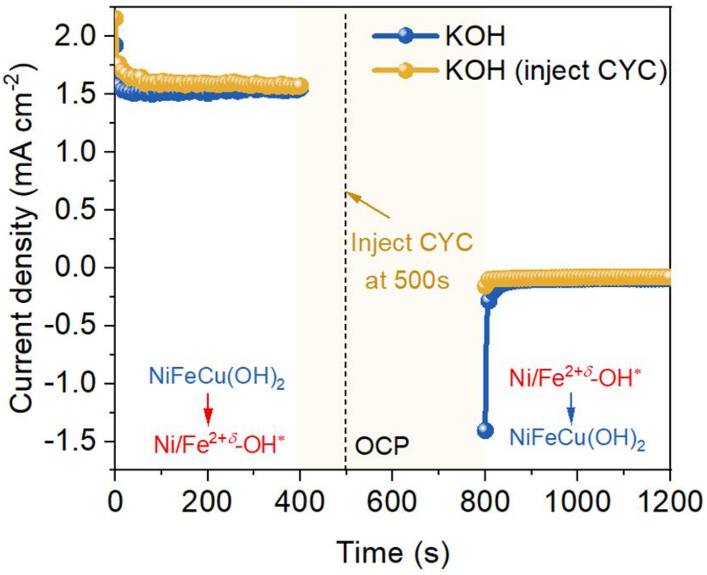

Fig. S47 Multi-potential step chronoamperometric measurement. Current density versus time for the $NiFeCu(OH)_2/TiO_2$ photoanode (0 to 400 s: 1.0 V vs. RHE; 400 to 800 s: open-circuit condition; 800 to 1200 s: 0.1 V vs. RHE) in 0.5 M KOH with 50 mM CYC.

When a potential of 1.0 V vs. RHE was applied, a positive current corresponding to the formation of $Ni/Fe^{2+\delta}$–OH* species was observed in the absence of CYC. Subsequently, a reduction potential was performed and an obvious negative current was generated, which can be assigned to the photoelectrochemical consumption of surface $Ni/Fe^{2+\delta}$–OH* species. After adding 50 mM CYC into the electrolyte, the reduction current disappeared under an open circuit conditions, indicating that the $Ni/Fe^{2+\delta}$–OH* is consumed by CYC via a spontaneous dehydrogenation process. These results further prove that the surface $Ni/Fe^{2+\delta}$–OH* species serve as the authentic active species for CYC oxidation.

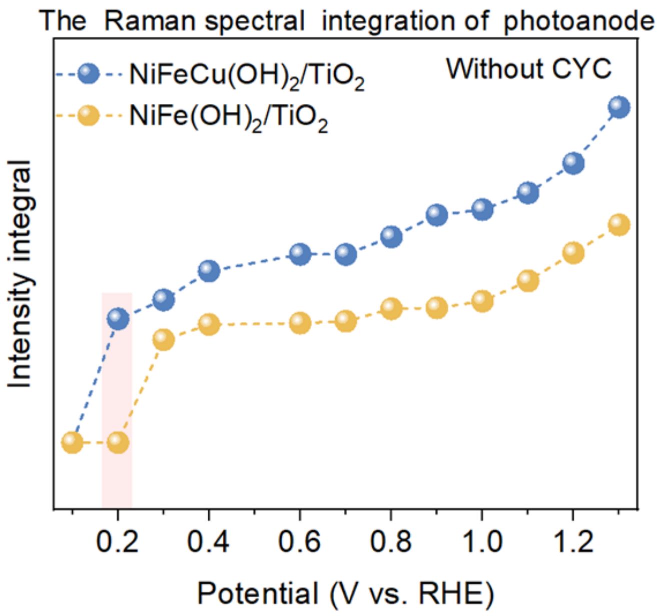

Fig. S48 *In-situ* Raman spectra intensity integral. Intensity integral plot of *in-situ* Raman spectral peaks of $NiFeCu(OH)_2/TiO_2$ and $NiFe(OH)_2/TiO_2$ in 0.5 M KOH.

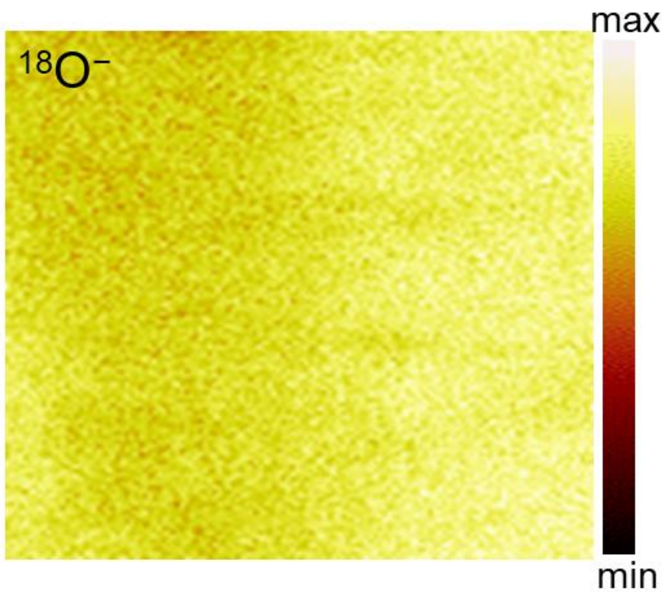

Fig. S49 TOF-SIMS 2D mapping. TOF-SIMS 2D mapping of $^{18}O^{-}$ negative secondary ions at the vicinity of the $NiFeCu(OH)_2/TiO_2$ surface after reaction in 0.5 M KOH solution containing 10% $H_2{}^{18}O$ at 1.0 V vs. RHE for 600 s.

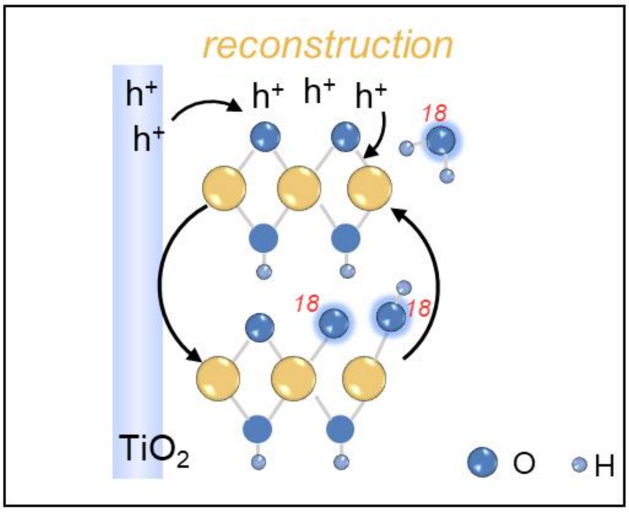

Fig. S50 Schematic illustration of Ni/Fe$^{2+\delta}$–OH*. Schematic illustration of the formation of Ni/Fe$^{2+\delta}$–OH* in NiFeCu(OH)$_2$/TiO$_2$ photoanode.

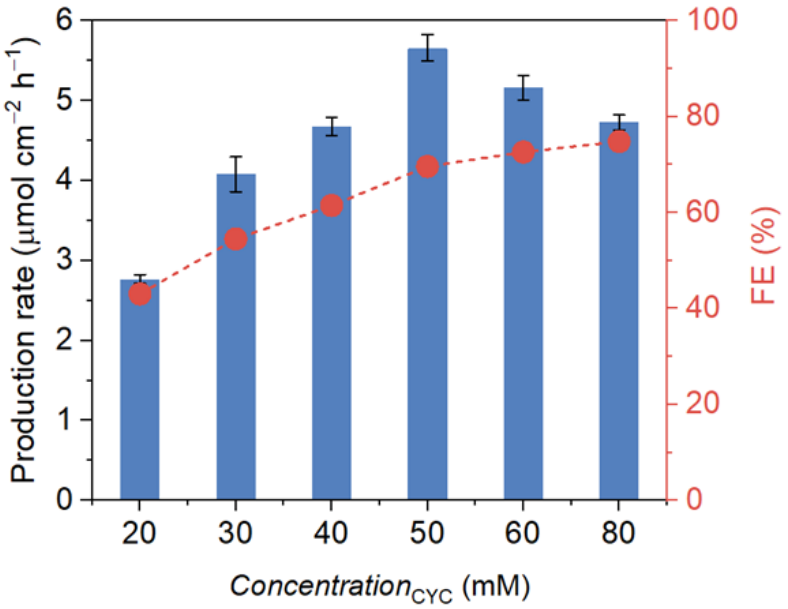

Fig. S51 The performance of PEC CYC oxidation at different CYC concentrations. Production rate of adipic acid with different concentrations of CYC over $NiFe(OH)_2/TiO_2$ and $NiFeCu(OH)_2/TiO_2$ photoanodes at 1.0 V vs. RHE under illumination.

We applied a constant potential of 1.0 V vs. RHE with varied CYC concentrations (20 ~ 80 mM) and fixed OH^- concentrations (0.5 M) over $NiFe(OH)_2/TiO_2$ and $NiFeCu(OH)_2/TiO_2$ photoanodes. It was observed that the adipic acid production rate of $NiFeCu(OH)_2/TiO_2$ significantly exceeds that of $NiFe(OH)_2/TiO_2$ across all applied CYC concentrations. More importantly, increasing the CYC concentration to 80 mM led to an enhanced adipic acid production rate for both $NiFeCu(OH)_2/TiO_2$ and $NiFe(OH)_2/TiO_2$. This result provides strong evidence for the superior adsorption properties and rapid mass transfer of the CYC substrate on the surface of the $NiFeCu(OH)_2/TiO_2$ photoanode.

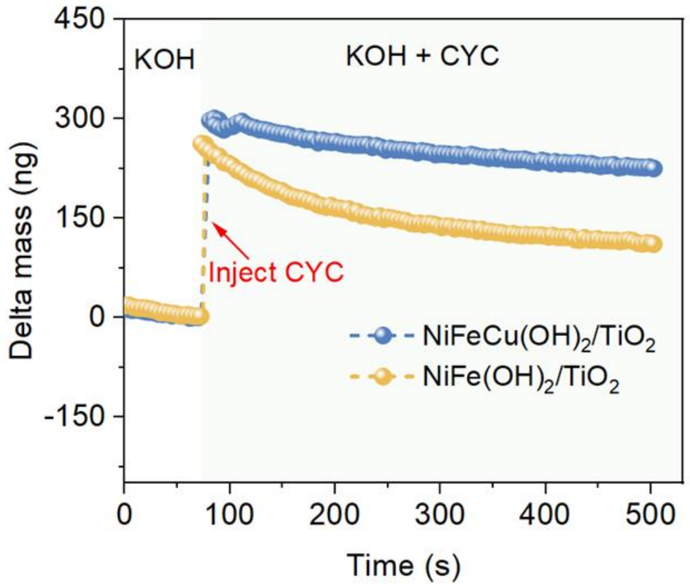

Fig. S52 QCM measurement. QCM mass response over $NiFe(OH)_2/TiO_2$ and $NiFeCu(OH)_2/TiO_2$ samples in 0.5 M KOH before and after adding 50 mM CYC.

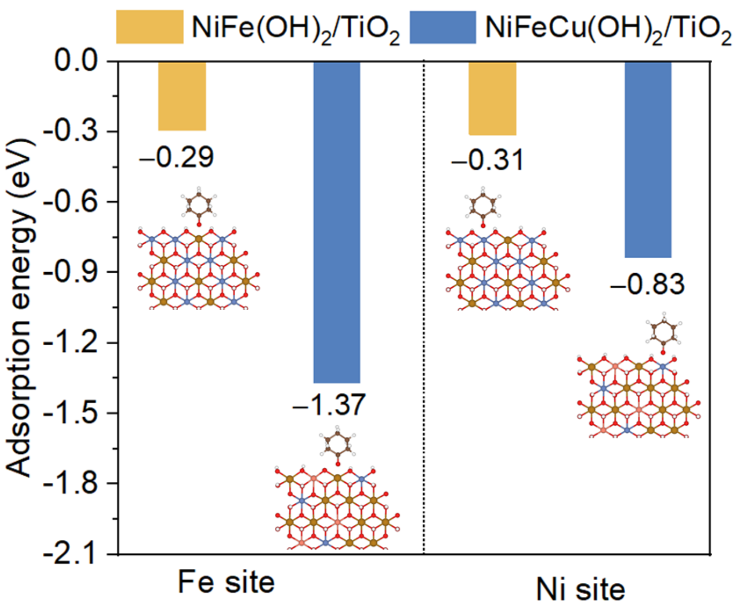

Fig. S53 DFT of strengthened CYC adsorption. Adsorption energies of CYC over $NiFe(OH)_2/TiO_2$ and $NiFeCu(OH)_2/TiO_2$. The optimized adsorption geometries are also displayed. The color of each element is blue for Ni, brown for Fe, pink for Cu, red for O, and white for H, respectively.

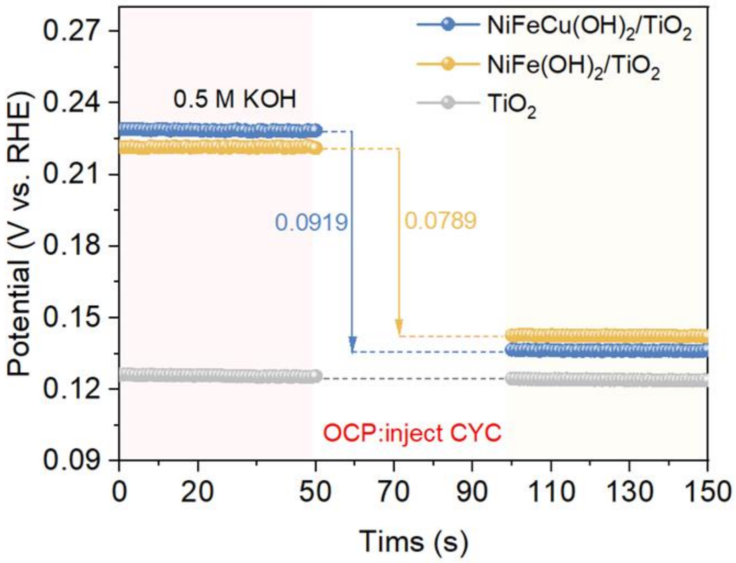

Fig. S54 Studies of strengthened CYC adsorption. OCP curves of TiO_2, $NiFe(OH)_2/TiO_2$, and $NiFeCu(OH)_2/TiO_2$ photoanodes in 0.5 M KOH with subsequent addition of 50 mM CYC.

The adsorption of CYC on photoanodes are studied by open circuit potential (OCP) tests. As shown in Fig. S54, after CYC introduction in KOH electrolyte, the TiO_2 exhibits almost no OCP shift, whereas $NiFe(OH)_2/TiO_2$ and $NiFeCu(OH)_2/TiO_2$ show a pronounced cathodic shift. The OCP decreases by 0.0919 V vs. RHE for $NiFeCu(OH)_2/TiO_2$ after adding 50 mM CYC in the electrolyte, much greater than that of $NiFe(OH)_2/TiO_2$ (0.0789 V vs. RHE), indicating a stronger adsorption of CYC after $NiFeCu(OH)_2$ modification.

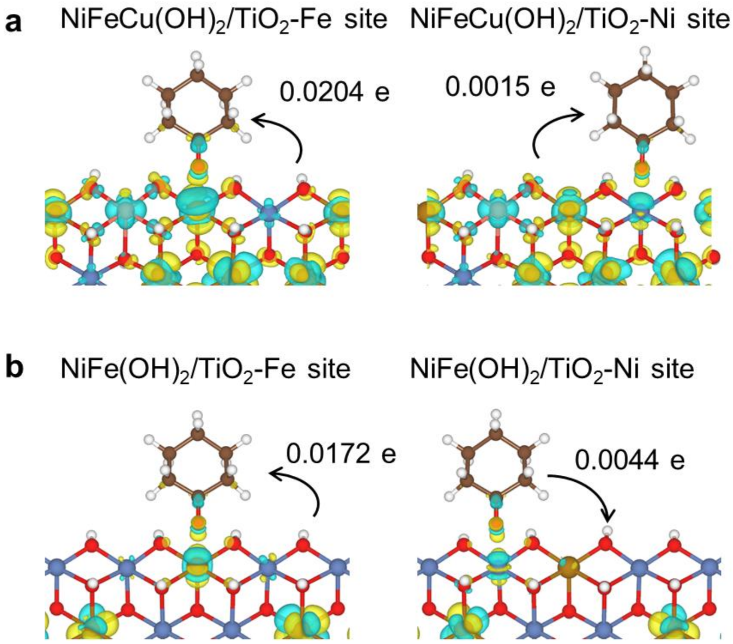

Fig. S55 Calculation. Charge density difference maps for CYC adsorption on (a) $NiFeCu(OH)_2/TiO_2$ (Fe and Ni sites) and (b) $NiFe(OH)_2/TiO_2$ (Fe and Ni sites). The color of each element is blue for Ni, brown for Fe, pink for Cu, red for O, and white for H, respectively.

To further elucidate the interaction between the catalyst surface and the organic substrate, charge density difference calculations were performed for cyclohexanone (CYC) adsorption on both Fe and Ni sites (Fig. 55). The results reveal a distinct preference for electron transfer at the Fe sites compared to the Ni sites. Specifically, the net electron transfer from the $NiFeCu(OH)_2/TiO_2$ surface to the adsorbed CYC molecule at the Fe site increases to 0.0204 e, surpassing the value of 0.0172 e observed in the $NiFe(OH)_2/TiO_2$. In contrast, Ni sites exhibit negligible charge transfer. These results were consistent with their surface electronic character in both photoanodes (Fig. 56). These findings indicate that Fe serves as the dominant active center for CYC activation, facilitating stronger adsorption and promoting subsequent catalytic transformation through efficient electron donation. Briefly, while OH* species preferentially bind to Ni sites, CYC molecules favor adsorption at Fe sites on the $NiFe(OH)_2/TiO_2$ photoanode. The incorporation of Cu enhances this site-specific behavior.

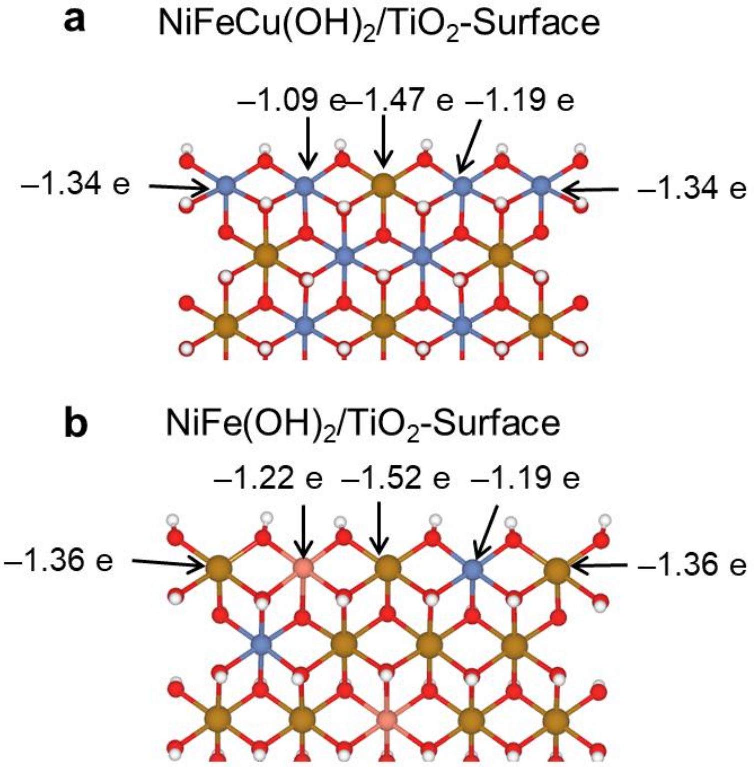

Fig. S56 Calculation. The surface electronic character in (a) $NiFeCu(OH)_2/TiO_2$ (Cu, Fe and Ni sites) and (b) $NiFe(OH)_2/TiO_2$ (Fe and Ni sites) photoanodes.

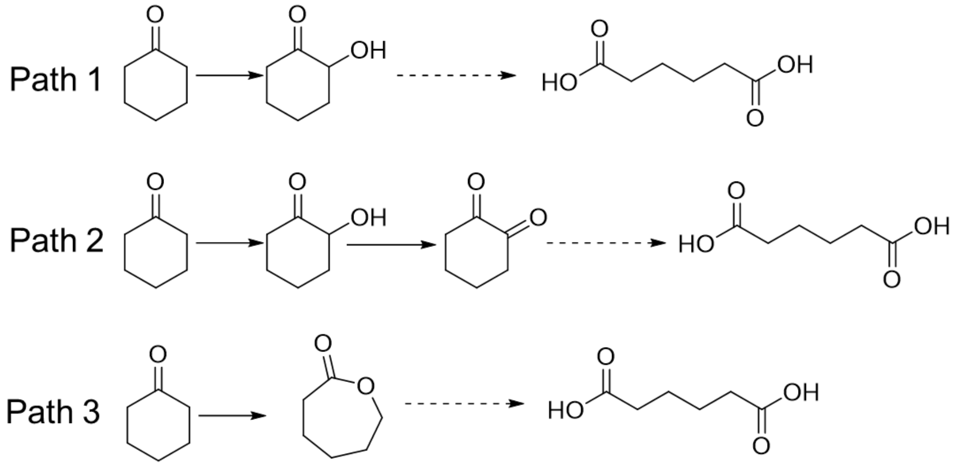

Fig. S57 Possible reaction pathways of CYC oxidation to adipic acid reported in the literature [9-12].

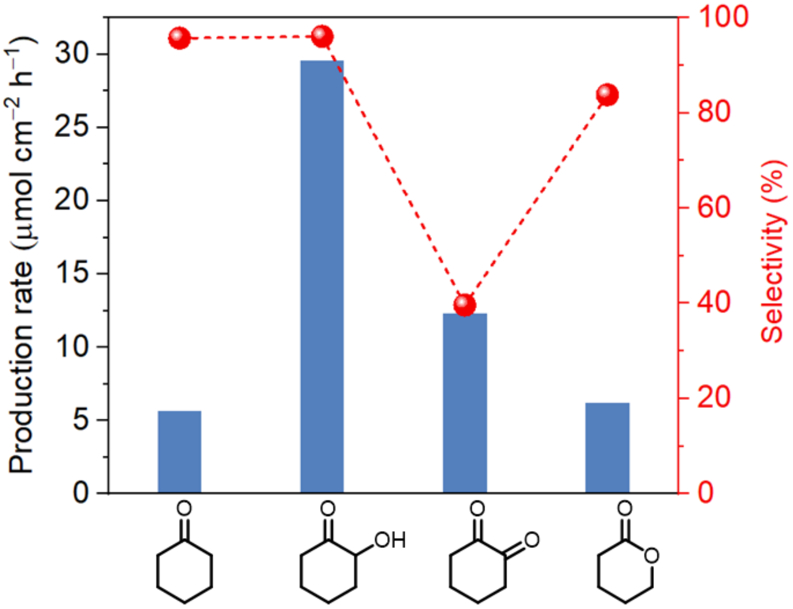

Fig. S58 The performance of PEC CYC oxidation over different possible intermediates. The product distribution for PEC oxidation of different possible intermediates over $NiFeCu(OH)_2/TiO_2$ photoanode at 1.0 V vs. RHE for 2 h.

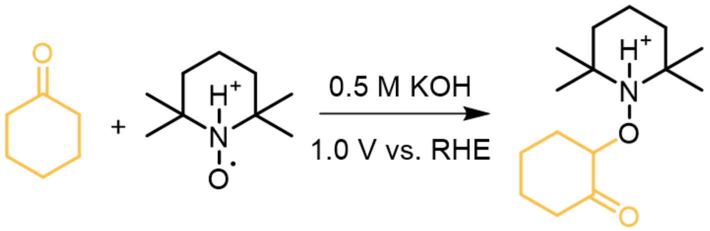

Fig. S59 Schematic illustration of TEMPO used as a radical trap.

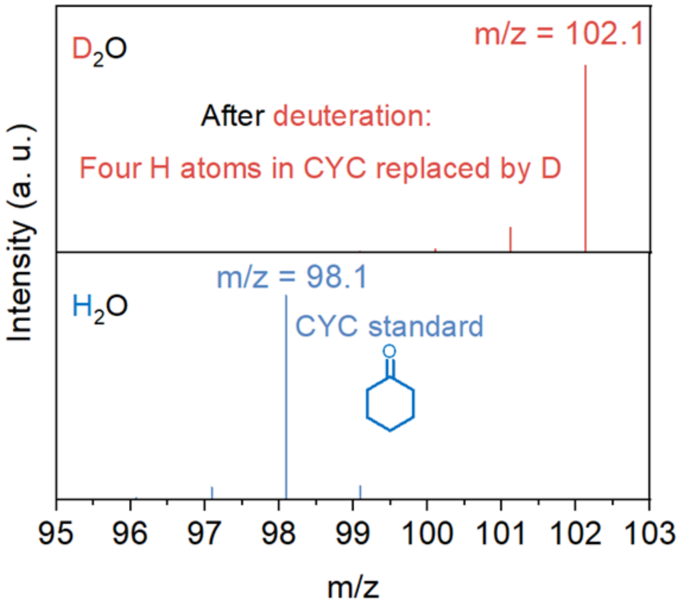

Fig. S60 GC-MS analysis. GC-MS spectra of the CYC in an isotope-labeled electrolyte with D_2O.

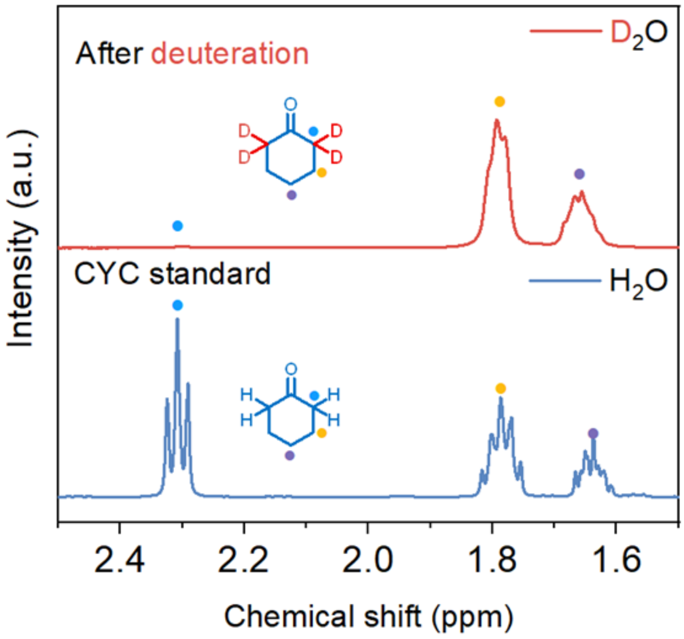

Fig. S61 ^{1}H NMR analysis. ^{1}H nuclear magnetic resonance (^{1}H NMR) of CYC and D4−deuterated CYC.

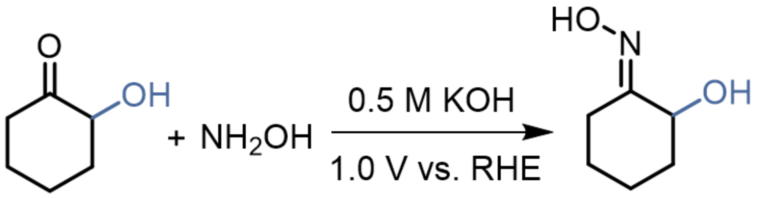

Fig. S62 Schematic illustration of NH_2OH used as a radical trap.

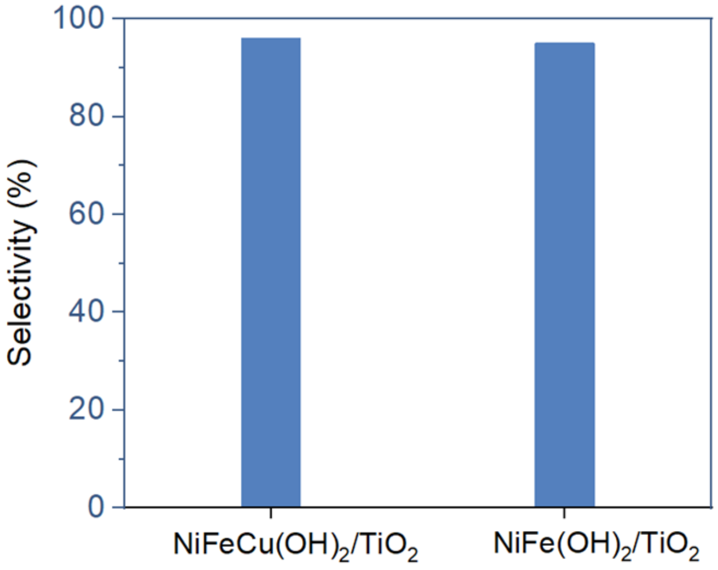

Fig. S63 The performance of PEC 2-hydroxycyclohexanone oxidation. The selectivity of adipic acid for PEC oxidation of 2-hydroxycyclohexanone intermediates over $NiFeCu(OH)_2/TiO_2$ photoanode at 1.0 V vs. RHE for 2 h.

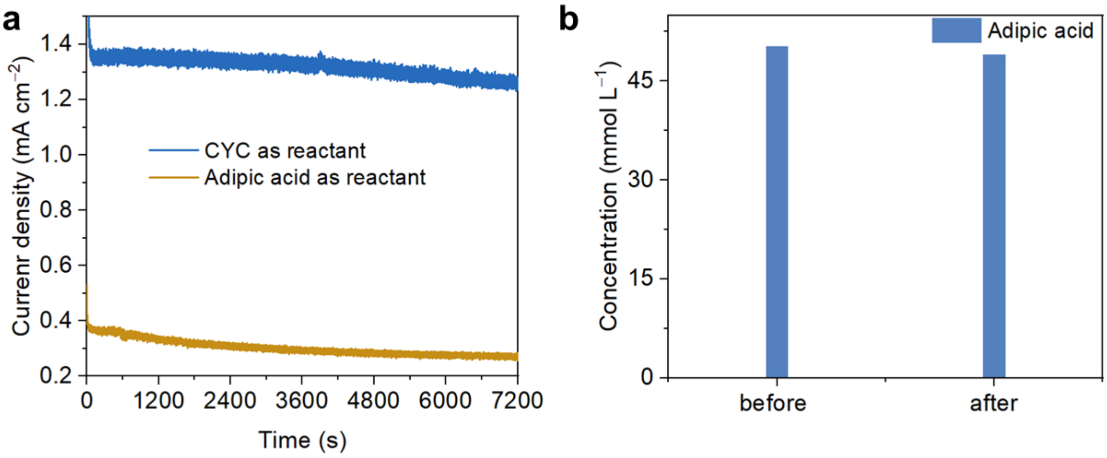

Fig. S64 The stability of adipic acid. (a) Current-time (*I*-*t*) curves of $NiFeCu(OH)_2/TiO_2$ photoanode in 0.5 M KOH with 50 mM CYC or adipic acid at 1.0 V vs. RHE under AM 1.5G (100 mW cm^{-2}) illumination. (b) The corresponding concentration of adipic acid before and after the PEC reaction

When adipic acid was used directly as a reactant, the current-time (*I*-*t*) curve shows a lower anodic photocurrent response compared to that of CYC oxidation (Fig. S64a), indicating that the further conversion of adipic acid is a kinetically unfavorable process. In addition, the HPLC results show that adipic acid can not convert into other by-products, such as glutaric acid (Fig. S64a). Crucially, the electron-withdrawing carbonyl group (C=O) in CYC markedly weakens the adjacent C_α–H bonds, rendering them acidic and highly susceptible to abstraction by Ni/$Fe^{2+\delta}$–OH*. Conversely, the carboxyl group (−COOH) in adipic acid provide insufficient activation for the remaining C−H bonds; the central methylene units retain strong, alkane-like character that resists cleavage under mild conditions. Additionally, the saturated C−C backbone of adipic acid is thermodynamically stable, lacking the ring strain or electronic predisposition required for bond scission. These results illustrate that adipic acid is relatively stable and can be accumulated over $NiFeCu(OH)_2/TiO_2$ photoanode in our system.

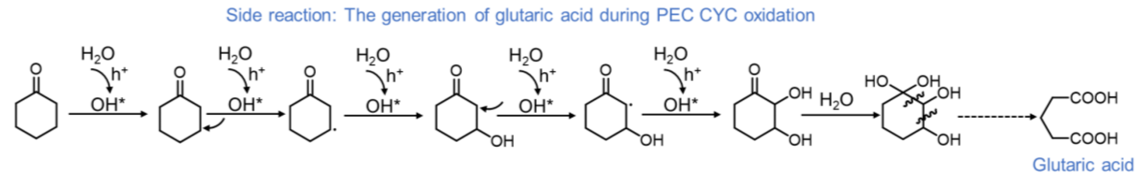

Fig. S65 A plausible reaction mechanism for the formation of glutaric acid as side product.

Supplementary Tables

Table S1. Ni/Fe/Cu ratio calculated for as-synthesized $NiFeCu(OH)_2/TiO_2$ based on ICP-OES analysis.

Element	Ni	Fe	Cu
content(mg/kg)	6.111	15.306	3.007

Table S2. Summary of the performances of the PEC CYC oxidation.

Catalyst	Electrolyte	Substrate	Light intensity	Bias	Production rate	selectivity	Ref
$Ni(OH)_2/TiO_2$	0.5 M KOH	CYC 50 mM	AM 1.5G (100 mW cm^{-2})	0.8 V vs. RHE	2.3 μmol cm^{-2} h^{-1}	90.6%	[13]
Ir_1/TFO	1 M KOH	CYC 20 mM	AM 1.5G (100 mW cm^{-2})	1.5 V vs. RHE<br>1.1 V vs. RHE	6.0 μmol cm^{-2} h^{-1}<br>~2.4 μmol cm^{-2} h^{-1}	88%<br>~70%	[14]
$NiFeCu(OH)_2$ $/TiO_2$	0.5 M KOH	CYC 50 mM	AM 1.5G (100 mW cm^{-2})	1.0 V vs. RHE	5.6 μmol cm^{-2} h^{-1}	95%	**This work**

Table S3. Investigation of the leaching of Cu^{2+} before and after reaction.

**Total Cu content (mg/L)** ^{b}	**Content of Cu in electrolyte** ^{c} **(mg/L) / Leaching ratio (%)**	
	**After 20 h reaction** ^{a}	**After 40 h reaction** ^{a}
3.007^{b}	0.006 / 0.21	0.009 / 0.29

^{a}Reaction conditions: $NiFeCu(OH)_2/TiO_2$ photoanode in 0.5 M KOH electrolyte with 50 mM

CYC under AM 1.5G illumination (100 mW cm^{-2});

^{b,c}Test conditions: ^{b}Agilent ICP-OES 725 ES, ^{c}Agilent ICP-MS 7800. RF Power: 1.20 kW, Plsama flow: 15.0 L/min, Nebulizer flow: 0.75 L/min, Sample uptake delay: 10 s, Replicate read time: 15 s, Replicates: 3.

Table S4 The distinction of PEC OLFB from (i) conventional redox flow batteries, (ii) light-assisted RFBs, and (iii) PEC organic oxidation systems.

	**RFBs**	**Light-assisted RFBs**	**PEC organic oxidation systems**	**PEC open-loop flow battery**
**Input energy**	Electricity	Solar energy	Solar energy	**Solar energy**
**Active species**	Reversible redox couples (*e.g.*, V^{3+}/V^{2+})	Reversible redox couples (*e.g.*, V^{3+}/V^{2+})	Organic substrates	**Organic substrates/ + Redox mediators**
**Output**	Electricity	Electricity	Chemical energy	**Chemical energy and electricity simultaneously**

As summarized in Table S4, unlike conventional or light-assisted RFBs that rely on reversible closed-loop cycles, the PEC OLFB operates in an open-loop mode. This configuration enables the co-generation of electricity and value-added chemicals from solar energy, distinguishing it fundamentally from standalone PEC organic oxidation systems which typically do not efficiently output electrical energy.

Supplementary Notes

Supplementary Note 1

As shown in Fig. S2, X-ray diffraction (XRD) patterns of TiO_2, $NiFe(OH)_2/TiO_2$ and the $NiFeCu(OH)_2/TiO_2$ samples reveal a typical rutile TiO_2 phase [1], consistent with the Raman results [15] (Fig. S3). It is noted that no diffraction peaks corresponding to $NiFe(OH)_2$ or $NiFeCu(OH)_2$ were detected due to the low loading. High-resolution TEM (HRTEM) images further reveal the core–shell structure of $NiFeCu(OH)_2/TiO_2$ sample, and the fringe distance of 0.321 nm corresponds well with the (110) facet of rutile TiO_2 (Fig. S4) [16]. Additionally, scanning transmission electron microscopy combined with energy-dispersive spectrometry (STEM-EDS) mapping results confirm the uniform distribution of Ni, Fe, and Cu elements throughout the TiO_2 nanorods (Fig. S5). Moreover, the atomic ratio of Ni/Fe/Cu in $NiFeCu(OH)_2$ is close to 1.0/4.0/0.7, and the content of Cu is detected by ICP-OES (Table S1).

X-ray photoelectron spectroscopy (XPS) was applied to investigate the surface elements and their chemical states of the as-prepared samples. The full XPS spectrum shows the presence of elements Ti, O, Ni, Fe, and Cu in $NiFeCu(OH)_2/TiO_2$ (Fig. S6). The pristine TiO_2 exhibits characteristic XPS peaks of Ti^{4+} at 458.7 and 464.4 eV, respectively [17]. As shown in Fig. S7, after modifying $NiFe(OH)_2$ with Cu, the Ti 2*p* peaks exhibit more negative shifts (~0.7 eV) compared to that in TiO_2, implying an enhanced electron transfer from $NiFeCu(OH)_2$ to TiO_2. The Ni 2p XPS spectra in $NiFe(OH)_2/TiO_2$ displays characteristic spin-orbit peaks of Ni^{2+} (fitting peak at 855.7 eV and satellite at 861.6 eV) [12]. After Cu modification, the Ni 2*p* peaks (855.8 eV vs 855.7 eV) showed no significant shift (Fig. S8a). The binding energies of Fe $2p_{3/2}$ in $NiFe(OH)_2/TiO_2$ sample was observed at 711.9 eV [18], corresponding to Fe^{3+}. Note that the Fe $2p_{3/2}$ peaks in $NiFeCu(OH)_2/TiO_2$ display positive shifts (0.9 eV) compared to that in $NiFe(OH)_2/TiO_2$, indicating the formation of electron-deficient Fe (Fig. S8b). The O 1*s* XPS spectrum of TiO_2 could be divided into two peaks, such that the peaks at 529.9 and 531.6 eV assigned to the lattice oxygen (O_L) and adsorbed oxygen species (O_{Ads}), respectively [15] (Fig. S9). The O 1*s* peaks of $NiFe(OH)_2/TiO_2$ sample appeared

at 529.7, 531.1 and 532 eV, corresponding to O_L, hydroxyl groups (NiFe-OH), and O_{Ads}, respectively (Fig. S10). After Cu modification, the Cu $2p_{3/2}$ peak appears which can be deconvoluted into peaks located at 934.6 eV assigned to Cu^{2+}, indicating that the Cu species exist predominantly in high oxidation states in the structure [9] (Fig. S11). This shift to lower energy for Ti^{4+} accompanied with the positive shift for Fe^{3+} is an indication that electron transfer from Fe to Ti, which can facilitate photogenerated electron-hole pairs separation, thereby improving PEC CYC oxidation performance.

Supplementary Note 2

To understand the foregoing promoted photocurrent densities of $NiFeCu(OH)_2/TiO_2$ photoanode for CYC oxidation, we evaluated the optical properties, charge separation and transfer kinetics of different photoanodes. UV−vis diffuse reflectance spectroscopy (DRS UV−vis) of the pristine TiO_2 photoanode shows an absorption edge at ~410 nm corresponding to the wide band gap of rutile TiO_2 (3.16 eV) [17] (Fig. S13). After the introduction of (oxy)hydroxide, the absorption edge of $NiFe(OH)_2/TiO_2$ and $NiFeCu(OH)_2/TiO_2$ did not obviously change, implying that light-harvesting efficiency is not a major factor in improving the PEC performance. To evaluate the charge injection efficiency (η_{inj}), we measured the PEC performance of these samples in the electrolyte with 0.5 M Na_2SO_3 (Fig. S14) [19]. The results show that the η_{inj} (72.5%) of $NiFeCu(OH)_2/TiO_2$ is higher than that of pristine TiO_2 (η_{inj} = 55.1%) and $NiFe(OH)_2/TiO_2$ (η_{inj} = 63.4%), implying the enhanced surface holes trapping and reaction kinetics after $NiFeCu(OH)_2$ modification (Fig. S15). In addition, the Mott-Schottky plots of three photoanodes that measured in the darkness show that the flat band potential of $NiFeCu(OH)_2/TiO_2$ exhibited a flatter slope, indicating that the introduction of $NiFeCu(OH)_2$ increases the carrier density, which is beneficial for bulk charge transport and separation (Fig. S16). The steady-state photoluminescence (PL) spectra demonstrates that the (oxy)hydroxide can significantly inhibit the photogenerated carrier recombination (Fig. S17). Furthermore, the photogenerated charge recombination kinetics were further analyzed using time-resolved transient

photoluminescence (TRPL). The obtained average charge lifetime follow the order: 6.21 ns > 6.08 ns > 5.8 ns, also suggesting that the introduction of $NiFe(OH)_2$ and $NiFeCu(OH)_2$ enhances hole utilization in the photoanode, leading to a slower charge recombination and thus extending the photoanode's lifetime (Fig. S18). Density of states (DOS) calculations further indicate that the introduction of Cu into $NiFe(OH)_2$ creates an empty band near the Fermi level (E_F), thereby promoting more efficient separation of photogenerated electron-hole pairs (Fig. S19). These results demonstrate that $NiFeCu(OH)_2$ modification can inhibit the photogenerated carrier recombination, while simultaneously promote carrier separation and transfer thus leading to higher photocurrent to a certain extent.

Supplementary Note 3

The stability of the $NiFeCu(OH)_2/TiO_2$ photoanode was evaluated by performing multiple cycles for PEC CYC oxidation. The reaction was conducted for 2 h per cycle, and the electrolyte was refreshed after each cycle to ensure the sufficient supply of CYC. The production rate and selectivity of adipic acid over $NiFeCu(OH)_2/TiO_2$ photoanode could be maintained after the 20th cycle for overall 40 h, demonstrating a good durability for PEC CYC oxidation (Fig. S26)

We have characterized the used $NiFeCu(OH)_2/TiO_2$ photoanode by SEM, XRD, HRTEM, and EDS mapping after the recycle stability test (40 h). As shown in Fig. S27a, the SEM image of used $NiFeCu(OH)_2/TiO_2$ photoanode shows that $NiFeCu(OH)_2$ nanosheets are uniformly covered on the TiO_2 nanorods array, showing no difference in morphology with fresh-$NiFeCu(OH)_2/TiO_2$ photoanode. XRD results show that the used $NiFeCu(OH)_2/TiO_2$ exhibits a typical rutile TiO_2 phase, confirming that the structure of photoanode remains stable after PEC CYC oxidation (Fig. S27b). HRTEM image of the used $NiFeCu(OH)_2/TiO_2$ (Fig. S27c) reveals that TiO_2 exhibits lattice fringe distances of 0.316 nm, which closely corresponds to the (110) plane. The corresponding EDS mapping confirms the coexistence of Ti, O, Ni, Fe, and Cu elements throughout the TiO_2 nanorods (Fig. S27d). These results suggest that the

$NiFeCu(OH)_2/TiO_2$ photoanode is relatively stable under the PEC CYC oxidation reaction conditions.

Supplementary Note 4

We characterized the post-$NiFeCu(OH)_2/TiO_2$ photoanode using XPS and ICP after a 40-hour reaction (reaction conditions: 0.5 M KOH with CYC at 1.0 V vs. RHE) to investigate the compositional stability and Cu leaching of $NiFeCu(OH)_2$. The Ti 2*p* peaks spectra show negligible differences, with the characteristic Ti^{4+} peaks consistently located at 458.7 and 464.4 eV (Fig. S28a). Likewise, the Ni 2*p* and Fe 2*p* peaks spectra s exhibit Ni^{2+} and Fe^{3+} species remain dominant in the post-$NiFeCu(OH)_2/TiO_2$ (Figs. S28b, c).

Consistent with the other metal centers, the Cu $2p_{3/2}$ peak spectra show no discernible shift, with the dominant Cu^{2+} peak retained at 934.6 eV (Fig. S28d), indicating the Cu component remains stable after PEC CYC oxidation. In addition, the ICP results (Table S3) show that the leaching ratio of Cu^{2+} was insignificant, showing ~0.21 % after 20 h and 0.29 % after 40 h, suggesting the Cu in $NiFeCu(OH)_2/TiO_2$ catalyst is relatively stable under the PEC reaction conditions.

Supplementary Note 5

Notably, in PEC OLFB system, solar energy serves as the primary driving force, which enables the conversion of light into electricity and value-added chemicals simultaneously. Under illumination, photogenerated holes migrate to the photoanode surface to oxidize CYC into adipic acid, while VO_2^+ is reduced to VO^{2+}on the other side. The reaction potential for the photoelectrooxidation of CYC to adipic acid is –0.64 V vs. SHE, which is significantly lower than the value of 0.71 V vs. SHE observed in the dark (Fig. S29a). Note that VO_2^+/VO^{2+} redox shows the positive potential of 1.12 V vs. SHE, implying a relatively larger discharge voltage in the PEC OLFB. Consequently, when coupling photo-driven CYC oxidation with the VO_2^+/VO^{2+} redox couple, a theoretical open-circuit voltage (OCV) of 1.76 V is predicted, notably higher

than the OCV of 0.38 V observed in the dark. The battery exhibited negligible discharge capability at current densities of 0.6-0.9 mA cm^{-2} under dark conditions. Furthermore, we measured the OCV of the designed PEC OLFB under both dark and illuminated conditions. Furthermore, experimental OCV measurements confirm that illumination induces a significant positive shift, rising from 0.36 V in the dark to 1.74 V under illumination (Fig. S29b). These results demonstrate that solar energy input is a prerequisite for the operation of the PEC OLFB system, where it is concurrently converted into electricity and chemicals.

Supplementary Note 6

A PEC open-loop flow battery is constructed with a $NiFeCu(OH)_2/TiO_2$ photoanode immersed in an aqueous solution of VO^{2+} and 2 M H_2SO_4, and a graphite felt counter electrode immersed in a 2 M H_2SO_4 solution, separated by a cation-conductive Nafion-117 membrane (Fig. S31). Upon illumination of the photoanode, electron–hole pair generation occurs in the $NiFeCu(OH)_2/TiO_2$, with holes migrating to the surface to oxidize VO^{2+} to VO_2^+. Meanwhile, H^+ is reduced to generate H_2 on the opposite side through a reaction.

Supplementary Note 7

Furthermore, we calculated the Gibbs free energy of OH* formation (ΔG_{OH*}) at both Ni and Fe sites for the $NiFe(OH)_2/TiO_2$ and $NiFeCu(OH)_2/TiO_2$ photoanodes (Fig. S40). Notably, the ΔG_{OH*} at the Ni site is significantly lower for $NiFeCu(OH)_2/TiO_2$ (0.16 eV) compared to $NiFe(OH)_2/TiO_2$ (1.28 eV), indicating that Cu incorporation facilitates OH* formation at Ni centers. In contrast, the Fe sites in both photoanodes exhibit comparable formation energies (0.59 eV vs. 0.57 eV), suggesting that Cu doping has a negligible effect on OH* binding at Fe sites. These theoretical findings align well with the experimentally observed enhanced OH* generation over the $NiFeCu(OH)_2/TiO_2$ photoanode.

To further elucidate the introduction of Cu promotes the adsorption of OH*, we

carried out charge density difference on the surfaces of $NiFe(OH)_2/TiO_2$ and $NiFeCu(OH)_2/TiO_2$ photoanodes, respectively. Charge density difference maps (Fig. S41) reveal that Cu incorporation induces significant electronic polarization specifically at the Ni sites upon OH* adsorption. In contrast to the diffuse charge redistribution observed in $NiFe(OH)_2/TiO_2$, the $NiFeCu(OH)_2/TiO_2$ system exhibits highly localized electron accumulation between the Ni center and the oxygen atom of OH*. This enhanced interfacial polarization promotes efficient hole capture and thermodynamically optimizes the Ni–OH* binding strength, consistent with the drastically reduced ΔG_{OH*} (from 1.28 to 0.16 eV). Density of states (DOS) analysis (Fig. S42) indicates that Cu incorporation upshifts the d-band center of Ni sites in $NiFeCu(OH)_2/TiO_2$ toward the Fermi level (E_F) relative to $NiFe(OH)_2/TiO_2$ (from −3.042 eV to −2.985 eV). According to d-band theory, this upshift strengthens the interaction between metal d-orbitals and OH* intermediates, thereby enhancing OH* adsorption. Thus, Cu doping promotes OH* formation and adsorption by optimizing the surface electronic structure.

Supplementary Note 8

The radical quenching experiments over $NiFeCu(OH)_2/TiO_2$ were performed in the presence of various radical scavengers (5 mM) for 2 h (TEMPO as radical scavenger, Na_2SO_3 as hole scavenger, Tert-Butanol (TBA) as OH* radical scavenger, $K_2S_2O_8$ as electron scavenger; Normal, no scavenger), the reaction was performed in 0.5 M KOH electrolyte with 50 mM cyclohexanone at 1.0 V vs. RHE under AM 1.5G illumination (100 mW cm^{-2}) for 2 h.

Supplementary Note 9

The isotope-labeling experiments using D_2O as the H source (0.5 M KOH was dissolved in D_2O) to examine the specific structure of C-centered radicals. Gas chromatography-mass spectrometry (GC-MS) results revealed that four H atoms in

CYC were replaced by D (Fig. S60). ^1H nuclear magnetic resonance (^1H NMR) spectrum gave more detailed information on the structure of deuterated CYC (Fig. S61). The peaks at 1.78 and 1.63 ppm in the ^1H NMR spectrum matched well with the standard CYC sample.

Supplementary References

1. Zhang R., *et al.* Photoelectrochemical catalysis toward selective anaerobic oxidation of alcohols. *Chem. Eur. J.* 2017; **23**: 8142−8147.
2. Kresse G., Furthmüller J. Efficiency of ab-Iinitio total energy calculations for metals and semiconductors using a plane-wave basis set. *Comput. Mater. Sci.* 1996; **6**: 15−50.
3. Kresse G., Hafner J. Ab initiomolecular dynamics for liquid metals. *Phys. Rev. B* 1993; **47**: 558−561.
4. Kresse G., Furthmüller J. Efficient iterative schemes for ab initio total-energy calculations using a plane-wave basis set. *Phys. Rev. B* 1996; **54**: 11169−11186.
5. Perdew J. P., Burke K., Ernzerhof M. Generalized gradient approximation made simple. *Phys. Rev. Lett.* 1996; **77**: 3865−3868.
6. Kresse G., Joubert D. From ultrasoft pseudopotentials to the projector augmented-wave method. *Phys. Rev. B* 1999; **59** 1758−1175.
7. Dudarev S. L., Botton G. A., Savrasov S. Y., Humphreys C. J., Sutton A. P. Electron-energy-loss spectra and the structural stability of nickel oxide: An LSDA+U Study. *Phys. Rev. B* 1998; **57**: 1505−1509.
8. Grimme S., Antony J., Ehrlich S., Krieg H. A consistent and accurateab initioparametrization of density functional dispersion correction (DFT-D) for the 94 elements H-Pu. *J. Chem. Phys.* 2010; **132**: 154104−154124.
9. Wang R., *et al.* Electrifying adipic acid production: Copper-promoted oxidation and C−C cleavage of cyclohexanol. *Angew. Chem. Int. Ed.* 2022; **61**: 1−9.
10. Li Z., *et al.* Electrocatalytic synthesis of adipic acid coupled with H_2 production enhanced by a ligand modification strategy. *Nat. Commun.* 2022; **13**: 5009−5021.

11. Jia Y., *et al.* Directional electrosynthesis of adipic acid and cyclohexanone by controlling the active sites on NiOOH. *J. Am. Chem. Soc.* 2024; **146**: 1282−1293.
12. X. Liu, *et al.* Electrosynthesis of adipic acid with high faradaic efficiency within a wide potential window. *Nat. Commun.* 2024; **15**: 7685−7701.
13. Zhang S., *et al.* Photoelectrochemical synthesis of adipic acid by selective oxidation of cyclohexanone. *Adv. Energy Mater.* 2025; **15**: 1−11.
14. Si S., *et al.* Selective photoelectrochemical synthesis of adipic acid using single-atom Ir decorated α-Fe_2O_3 photoanode. *Nat. Commun.* 2025; **16**: 5128−5139.
15. Ning F., *et al.* TiO_2/graphene/NiFe-layered double hydroxide nanorod array photoanodes for efficient photoelectrochemical water splitting. *Energy Environ. Sci.* 2016; **9**: 2633−2643.
16. Luo L., *et al.* Selective photoelectrocatalytic glycerol oxidation to dihydroxyacetone via enhanced middle hydroxyl adsorption over a Bi_2O_3-incorporated catalyst. *J. Am. Chem. Soc.* 2022; **144**: 7720−7730.
17. Luo L., *et al.* Photoelectrocatalytic activation of C−H bond in toluene by titanium dioxide-supported subnanometric PtO_x clusters. *Angew. Chem. Int. Ed.* 2025; **64**: 1−11.
18. Tang J., *et al.* Local charge modulation induced the formation of high-valent nickel sites for enhanced urea electrolysis. *Adv. Energy Mater.* 2024; **14**: 2403004−2403012.
19. Liu B., *et al.* A standalone bismuth vanadate-silicon artificial leaf achieving 8.4% efficiency for hydrogen production. *Nat. Commun.* 2025; **16**: 2792−2805.
